# Structural Diversity and Tunable Emission in Hybrid Organic–Inorganic Copper(I) Bromides

**DOI:** 10.1021/acs.inorgchem.5c04578

**Published:** 2025-12-08

**Authors:** Tamanna Pinky, Kanika Parashar, Dilruba A. Popy, Kowsik Ghosh, Aleksandra D. Valueva, Mario F. Borunda, Svilen Bobev, Bayram Saparov

**Affiliations:** a Department of Chemistry & Biochemistry, 6187 University of Oklahoma, Norman, Oklahoma 73019, United States; b Department of Chemistry & Biochemistry, 5972 University of Delaware, Newark, Delaware 19716, United States; c Department of Chemistry, 1355 University of Georgia, Athens, Georgia 30602, United States; d Department of Physics, 7618 Oklahoma State University, Stillwater, Oklahoma 74078, United States

## Abstract

Recently, hybrid organic–inorganic copper­(I) metal halides have attracted global attention due to their intriguing optical properties and low-cost solution processability. In this work, we report three hybrid organic–inorganic copper­(I) bromides, [TMPA]_2_[Cu_2_Br_4_], [TMPA]_4_[Cu_6_Br_10_], and [TMPA]_2_[Cu_4_Br_6_], synthesized through a slow evaporation method using trimethylphenylammonium (TMPA^+^) as the organic cation. By precise control of the CuBr and TMPABr precursors, different copper halide [Cu_2_Br_4_]^2–^, [Cu_6_Br_10_]^4–^, and [Cu_4_Br_6_]^2–^ structural units can be obtained. [TMPA]_2_[Cu_2_Br_4_], [TMPA]_4_[Cu_6_Br_10_], and [TMPA]_2_[Cu_4_Br_6_] demonstrate distinct blue, orange, and greenish-yellow light emission, respectively. The first two compounds have zero-dimensional (0D) crystal structures in centrosymmetric triclinic space group *P*-1 and monoclinic space group *P*2_1_/*n*. In contrast, [TMPA]_2_[Cu_4_Br_6_] features a unique one-dimensional (1D) structure and crystallizes in the centrosymmetric monoclinic space group *P*2_1_/*c*. Consequently, the observed greenish-yellow emission of [TMPA]_2_[Cu_4_Br_6_] is also unique, in contrast to the typical orange-red emission of 0D [Cu_4_Br_6_]-based compounds. This work provides insights into the design of copper halide light emitters and emphasizes the influence of structural dimensionality on photoluminescence. The tunable optical properties suggest the potential of these materials for multicolor photopatterning, information encryption, and anticounterfeiting applications.

## Graphical abstract

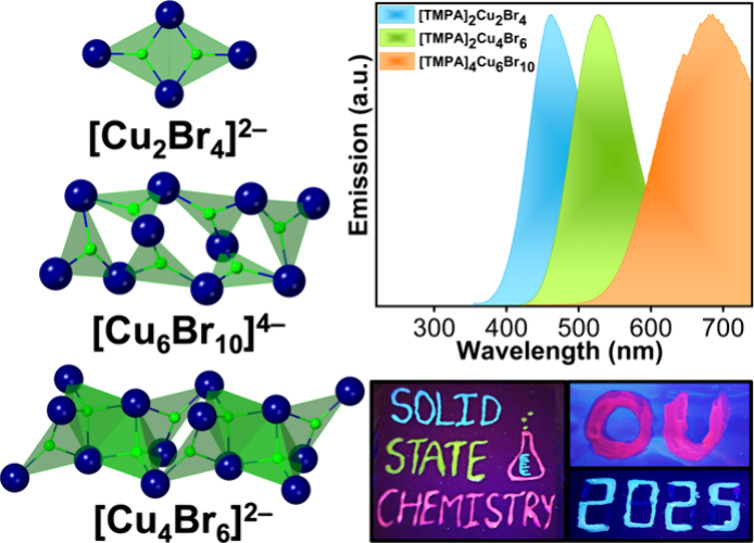

## 1 Introduction

Hybrid organic–inorganic Cu­(I) halides have recently become an important optical materials class due to their low toxicity, cost-effective solution processability, and outstanding photophysical properties.^1–9^ These materials are now recognized for their structural diversity and tunable luminescent properties, which make them suitable for various applications, including radiation detection, anticounterfeiting, and light-emitting diodes (LEDs).^1–3,10–16^ Examples of exciting Cu­(I) halide materials include (TMA)_3_Cu_2_Br_5_, (TEA)_2_Cu_2_Br_4_, and (TPA)_2_Cu_4_Br_6_,^1,3^ which have been reported to exhibit unique low-dimensional crystal structures that are capable of forming self-trapped excitonic (STE) states at room temperatures; photoemission from the STE states results in record light emission efficiency values up to 100%.^1–3,10–12,17,18^

Recent studies on hybrid organic–inorganic Cu­(I) halides demonstrate that the structurally distinct organic cations can facilitate the formation of different coordination environments around individual Cu­(I) centers, including linear, trigonal planar, and tetrahedral geometries, as well as unique combinations of these.^1,3,6,7,17,18^ In addition to influencing the coordination environment, these distinct organic cations are also known to cause fine changes in the crystal structure, including adjustments to bond lengths and bond angles.^1,10,17,19^ As a consequence, these fine structural modifications lead to observable differences in the optical properties, including in the emission colors of the materials.^1,6,18^ For instance, (TEA)_2_Cu_2_Br_4_ (TEA^+^ = tetraethylammounium) displays blue emission, whereas (TEP)_2_Cu_2_Br_4_ (TEP^+^ = tetraethylphosphonium) shows bright broad-band greenish-white emission.^1,17,20^ These compounds have similar crystal and electronic structures. The band structures suggest that Cu 3d orbitals and Cu 4s orbitals are the major contributors to the valence band maxima (VBM) and conduction band minima (CBM), respectively.^1,17,20^ Moreover, each Cu­(I) atom of the [Cu_2_Br_4_]^2–^ anion is coordinated to three bromide atoms in a trigonal planar arrangement in both compounds.^17,20,21^ Despite the overall structural similarity, an observation can be made that the [Cu_2_Br_4_]^2–^ anions are slightly different in these compounds. The Cu···Cu interatomic distance in (TEA)_2_Cu_2_Br_4_ is 2.937(3) Å, while it is 2.870(5) Å in (TEP)_2_Cu_2_Br_4_.^17,20,21^ The Br–Cu–Br bond angles are similar but slightly different, ranging from 106.3(1)–127.9(1)° in (TEA)_2_Cu_2_Br_4_ to 107.6(1)–128.2(1)° in (TEP)_2_Cu_2_Br_4_.^17,20,21^ Even though these distances and angles are only slightly different, it is believed that this is the origin of the observed optical property differences.^1,6^ Indeed, the previous studies on all-inorganic Cu­(I) halides CsCu_2_X_3_ (X = Cl, Br, and I) have shown that minor structural changes caused by halogen substitution can lead to stabilization of different STE states and a broad range of emission wavelengths from green to yellow.^22–24^ CsCu_2_X_3_ and other Cu­(I) halides with close Cu···Cu distances (i.e., less than the sum of the van der Waals radii of two Cu atoms (2.80 Å)) are known to exhibit photophysical properties that are sensitive to the precise value of these Cu···Cu distances.^1,6,22^ However, for most luminescent Cu­(I) halide material families, only limited studies have been conducted; an in-depth understanding of the relationship between Cu···Cu interatomic distances and photophysical properties of these is yet to be established.

Recent research on hybrid Cu­(I) halides has also shown that with different inorganic structural units such as [CuX_2_], [Cu_2_X_4_], and [Cu_4_X_6_] (X = Br) can be stabilized using the same organic cations.^17,25–28^ However, there have been limited studies on the differences in optical properties among the compounds featuring different Cu–X inorganic units. Herein, we report syntheses, crystal structures, and photophysical properties of three structurally diverse hybrid organic–inorganic Cu­(I) bromides, [TMPA]_2_[Cu_2_Br_4_], [TMPA]_4_[Cu_6_Br_10_], and [TMPA]_2_[Cu_4_Br_6_] (TMPA^+^ = trimethylphenylammonium, [N­(C_6_H_5_)­(CH_3_)_3_]^+^). These compounds share the same organic cations TMPA^+^ but significantly differ in their copper-bromide inorganic polyanions. The crystal structures of [TMPA]_2_[Cu_2_Br_4_] and [TMPA]_4_[Cu_6_Br_10_] were originally reported in the early 1990s,^29–31^ whereas the unique one-dimensional (1D) structure of [TMPA]_2_[Cu_4_Br_6_] is reported here for the first time. Our optical spectroscopy measurements suggest that [TMPA]_2_[Cu_2_Br_4_], [TMPA]_4_[Cu_6_Br_10_], and [TMPA]_2_[Cu_4_Br_6_] emit in the blue, orange, and greenish-yellow regions, respectively. The obtained X-ray crystallography and optical spectroscopy results are discussed, together with the results of density functional theory (DFT) calculations.

## 2 Experimental Section

### 2.1 Materials

Copper­(I) bromide (CuBr, Sigma-Aldrich, > 99%), trimethylphenylammonium bromide ([N­(C_6_H_5_)­(CH_3_)_3_]­Br, TCI America, > 98.0%), absolute ethanol (EtOH, Pharmco, 99%), hypophosphorous acid (H_3_PO_2_) (Sigma-Aldrich, 50%), and hydrobromic acid (HBr, 48% in water, Sigma-Aldrich) were used as they were purchased without any additional purification. Unless specifically noted, all experimental synthesis work was performed in a nitrogen-filled glovebox.

### 2.2 [TMPA]_2_[Cu_2_Br_4_] Single Crystal Growth Using the Slow Evaporation Method

To synthesize [TMPA]_2_[Cu_2_Br_4_] single crystals, 1 mmol of trimethylphenylammonium bromide (TMPABr) and 1 mmol of CuBr were added in a glass vial. Then, 4 mL of EtOH, 0.6 mL of HBr, and 0.1 mL of H_3_PO_2_ are added to dissolve the precursor materials. The resultant mixture was stirred continuously and heated at 80 °C until complete dissolution occurred. In the following step, the resultant solution was allowed to cool to room temperature. After 2 days, when complete solvent evaporation was done, colorless block crystals were collected. The resulting crystals are 0.2 cm (approximate) in size. For further property measurements, we stored this crystal inside a nitrogen-filled glovebox.

### 2.3 [TMPA]_4_[Cu_6_Br_10_] Single Crystal Growth Using the Slow Evaporation Method

Single crystals of [TMPA]_4_[Cu_6_Br_10_] were prepared by combining 2 mmol of trimethylphenylammonium bromide (TMPABr) and 3 mmol of CuBr in a vial. To dissolve this mixture, 5 mL of EtOH, 0.6 mL of HBr, and 0.2 mL of H_3_PO_2_ were added. For complete dissolution, the mixture was stirred and heated at 80 °C. Thin plate-like transparent crystals (approximately 0.5 cm in size) were carefully harvested from the solution and stored in a nitrogen-filled glovebox for subsequent property measurements. [TMPA]_4_[Cu_6_Br_10_] can be synthesized using stoichiometric amounts of TMPABr and CuBr in a 1:1 ratio, followed by the same synthesis process. However, in this case, the crystals should be collected after 1 day of the reaction setup. If the solvent is allowed to fully evaporate, then [TMPA]_2_[Cu_2_Br_4_] forms as a product instead.

### 2.4 [TMPA]_2_[Cu_4_Br_6_] Single Crystal Growth Using the Slow Evaporation Method

Single crystals of [TMPA]_2_[Cu_4_Br_6_] were grown by dissolving 2 mmol of trimethylphenylammonium bromide (TMPABr) and 4 mmol of CuBr in 8 mL of EtOH with the addition of 0.6 mL of HBr and 0.2 mL of H_3_PO_2_. This followed the same procedure as mentioned for the other two compounds. The resulting colorless block crystals, approximately 0.2 cm in size, were stored in a nitrogen-filled glovebox for further measurements.

### 2.5 Luminescent Ink Preparation Using [TMPA]_2_[Cu_2_Br_4_], [TMPA]_4_[Cu_6_Br_10_], and [TMPA]_2_[Cu_4_Br_6_]

Luminescent inks of [TMPA]_2_[Cu_2_Br_4_], [TMPA]_4_[Cu_6_Br_10_], and [TMPA]_2_[Cu_4_Br_6_] were prepared by adding 2 g of poly­(methyl methacrylate) (PMMA) into 6 mL of toluene. The mixture was stirred at room temperature until PMMA dissolved completely. Then, 300 mg of microcrystalline powder samples of the title compounds were dispersed in the PMMA solution and stirred overnight. Eventually, these luminescent inks were utilized for glass painting, printing the latent “Butterfly” and “Solid State Chemistry” patterns, and encrypted patterns for anticounterfeiting applications.

### 2.6 Powder X-ray Diffraction (PXRD) Measurements

To perform the PXRD measurements, the Rigaku MiniFlex600 system was used. This system used a Cu Kα radiation source (Ni-filtered). All measurements were performed at room temperature on finely ground powders of single crystals. The PXRD scans were set in the 3–90° (2θ) range, with a step size of 0.02°. For data processing, the software package PDXL2 was used, and the resultant XRD patterns were fitted by the decomposition method.

### 2.7 Single Crystal X-ray Diffraction (SCXRD) Measurements

SCXRD data for [TMPA]_2_[Cu_2_Br_4_] and [TMPA]_4_[Cu_6_Br_10_] were collected using a Bruker D8 Quest diffractometer configured with Kappa-geometry. An Incoatec Iμs microfocus Mo Kα X-ray source was fitted to the diffractometer. For data acquisition, a Photon II area detector was used. By using equivalent reflections, the semiempirical technique did the absorption corrections. The APEX3 v2015.5-2 program has embedded software intrinsic phasing, which was used to determine the crystal structures. SCXRD data for [TMPA]_2_[Cu_4_Br_6_] were collected using suitable single crystals that were selected and cut under dry Paratone-N oil to appropriate dimensions (≤0.10 mm). After that, crystals were scooped by MiTeGen plastic loops and transferred to the goniometer of a Bruker APEX diffractometer. The experiments were done at a temperature of 100(2) K, which was maintained by a stream of cold nitrogen gas. Monochromatized Mo Kα radiation with λ = 0.71073 Å was used for the measurements. Data were processed with the programs from APEX3 software package (v2019. 1–0). Absorption corrections based on equivalent reflections were applied with SADABS. The crystal structures were solved using the intrinsic phasing method, implemented in SHELXT,^32^ and refined by the full-matrix least-squares method on *F*2 with SHELXL.^33^ Details of the data collection and structure refinement parameters are provided in Table S1. The Supporting Information includes atomic coordinates, equivalent isotropic displacement parameters, and selected bond lengths and angles. The Crystallographic Information Files (CIFs) have been deposited in the Cambridge Crystallographic Data Centre (CCDC) under deposition numbers 2481513–2481515.

### 2.8 Differential Scanning Calorimetry (DSC) and Thermogravimetric Analysis (TGA) Measurements

A TA Instruments SDT650 unit was used to perform the DSC and TGA measurements. Each sample was taken in 90 μL alumina crucibles and weighed approximately 10 mg. For measurements, the nitrogen flow rate was maintained at 100 mL/min and the temperature in a range of 30–475 °C with a heating rate of 10 °C/min. To determine the DSC onset temperatures, the TRIOS software analysis package was used.

### 2.9 Photoluminescence Measurements

Photoluminescence excitation (PLE) and emission (PL) measurements of single crystals and polycrystalline powder samples were performed at room temperature. To perform this measurement, a HORIBA Jobin Yvon Fluorolog-3 spectrofluorometer was used. This spectrofluorometer is equipped with a xenon lamp source and a Quanta-Φ integrating sphere. A two-curve method over a varied range of wavelengths from 250 to 750 nm was utilized for data acquisition.

### 2.10 Diffuse Reflectance Measurements

Optical band gap energy was studied by using a PerkinElmer Lambda 750 UV–vis-NIR spectrometer. This system used an InGaAs Integrating Sphere and was equipped with a 100 mm Spectralon. To perform the measurements, the 250–1100 nm wavelength range was covered by the spectrometer. In this work, we used the Kubelka–Munk function to convert diffuse reflectance into pseudoabsorption spectra. The function is generally represented as F(R)=αS=(1−R)22R , where α stands for absorption coefficient, *S* for scattering coefficient, and *R* for reflectance.

### 2.11 Density Functional Theory (DFT) Calculations

All of the electronic band structure and projected density of states (PDOS) calculations were carried out in the SIESTA software package.^34,35^ The density functional theory (DFT) calculations were done at the GGA level using the Perdew–Burke–Ernzerhof (PBE) and the PBEsol exchange correlation functionals.^36,37^ The pseudopotentials used were scalar relativistic with stringent norm-conserving as obtained from the PseudoDojo repository in PSML format.^38,39^ The cutoff energy used was 350 Ry, and we sampled the Brillouin zone using a 4 × 4 × 4 Monkhorst-Pack grid.^40^ The lattice parameters used in the DFT calculations were fixed to those obtained from XRD in all three systems. The relaxed atomic positions were found by minimizing the forces according to the conjugate gradient optimization method until all of the atomic forces were less than 0.052 eV/Å. The PDOS calculations used a Gaussian broadening of 0.1 eV. In addition, the electronic band structure of [TMPA]_2_[Cu_4_Br_6_] was calculated using the Vienna Ab Initio Package (VASP) planewave code,^41,42^ generalized gradient approximation of Perdew, Burke and Ernzerhof (PBE),^36^ and projector-augmented wave (PAW) method.^43^ The initial unit cells were converted to a primitive cell using VESTA software before geometry optimization.^44^ The ground-state geometries at 0 K were optimized by relaxing the cell volume, atomic positions, and cell symmetry until the maximum force on each atom is less than 0.01 eV/Å. Nonspin-polarized calculations were performed, with 520 eV cutoff energy for the plane wave basis set and 10^–5^ eV energy convergence criteria. The *k*-paths for band structure calculations were generated using VASPKIT package.^45^

## 3 Results and Discussion

Colorless single crystals of [TMPA]_2_[Cu_2_Br_4_], [TMPA]_4_[Cu_6_Br_10_], and [TMPA]_2_[Cu_4_Br_6_] were synthesized via the slow cooling method by controlling the ratio of the reactants (Figure 1). The obtained crystals are transparent under ambient light, suggesting that these compounds may exhibit minimal absorption in the visible region and have wide optical band gaps (Figure 1 and Figure S1). At room temperature, when illuminated with 365 nm UV light, they emit blue, orange, and greenish-yellow light (Figure 1).

**Figure 1:**
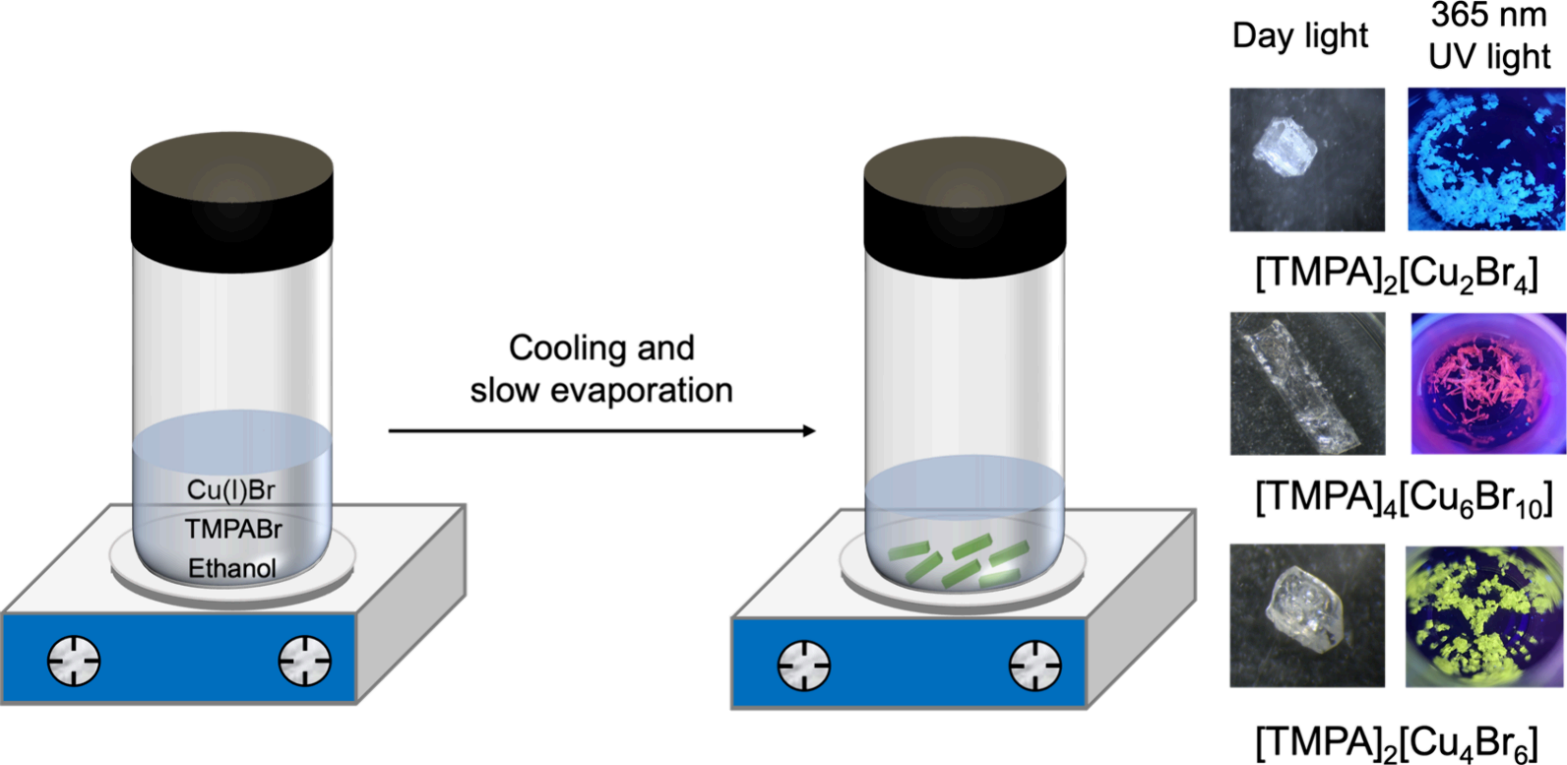
Schematic illustration of the synthesis procedure of [TMPA]_2_[Cu_2_Br_4_], [TMPA]_4_[Cu_6_Br_10_], and [TMPA]_2_[Cu_4_Br_6_] and optical photograph (under daylight and 365 nm UV light).

The crystal structures of the title compounds are shown in Figure 2, and the results of the SCXRD measurements are provided in Tables S1–S5. [TMPA]_2_[Cu_2_Br_4_] and [TMPA]_4_[Cu_6_Br_10_] crystallize in centrosymmetric triclinic space group *P*-1 and monoclinic space group *P*2_1_/*n*, respectively. The SCXRD results obtained for [TMPA]_2_[Cu_2_Br_4_] in this work are in agreement with the earlier report in 1980s on this compound.^31^ Later studies in this pseudoternary system showed the presence of other phases in addition to the earlier reported [TMPA]_2_[Cu_2_Br_4_].^31^ A decade later, another product was obtained and reported as [TMPA]_4_[Cu_6_Br_10_].^30^ During our synthesis of [TMPA]_2_[Cu_2_Br_4_], we observed the initial growth of plate-like thin crystals on the first day of the reaction, which eventually transformed into block-like crystals of the target phase [TMPA]_2_[Cu_2_Br_4_]. Analysis of the plate-like crystals via SCXRD measurements confirmed them to be [TMPA]_4_[Cu_6_Br_10_]. This compound can also be directly synthesized by combining stoichiometric amounts of trimethylphenylammonium bromide (TMPABr) and CuBr (see Section 2). In contrast, [TMPA]_2_[Cu_4_Br_6_] is a new compound reported here for the first time. It crystallizes in the centrosymmetric monoclinic space group *P*2_1_/*c*. Powder X-ray diffraction experiments performed at room temperature confirmed the phase purity and crystallinity of all three compounds (Figure 3).

**Figure 2:**
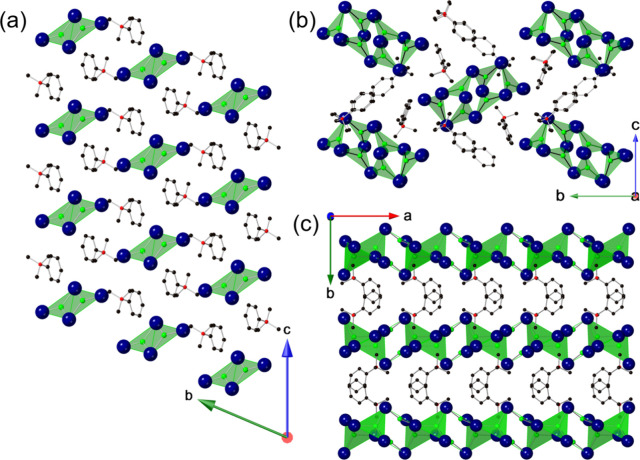
Crystal structures of (a) [TMPA]_2_[Cu_2_Br_4_], (b) [TMPA]_4_[Cu_6_Br_10_], and (c) [TMPA]_2_[Cu_4_Br_6_]. Hydrogens have been omitted for clarity. Green, blue, red, and black spheres represent Cu, Br, N, and C atoms, respectively.

**Figure 3:**
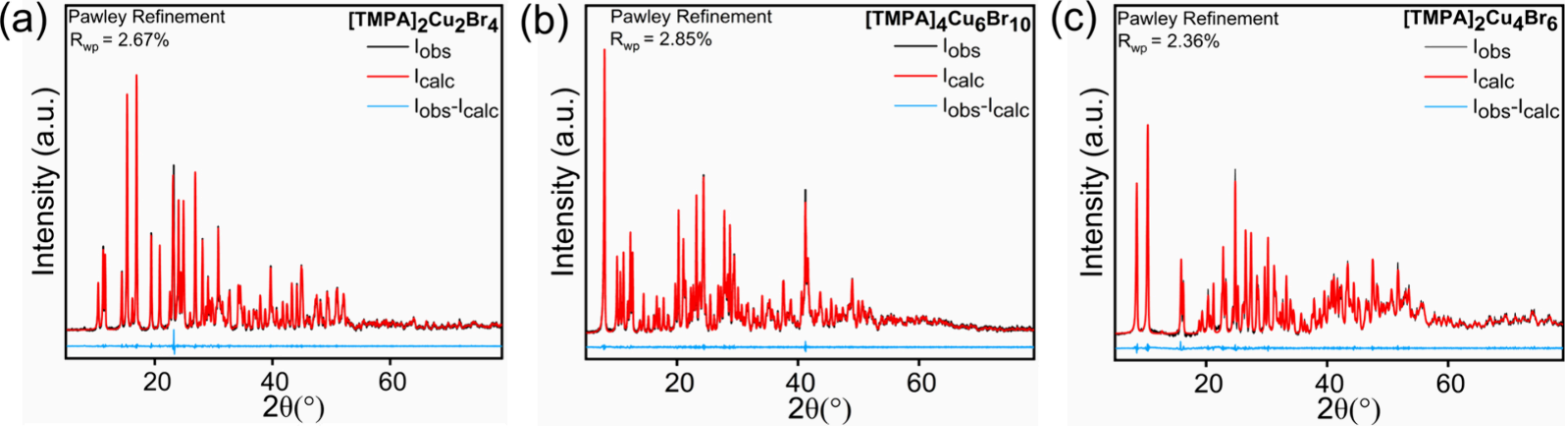
Room-temperature PXRD patterns (in black) fitted using the Pawley method (in red) for (a) [TMPA]_2_[Cu_2_Br_4_], (b) [TMPA]_4_[Cu_6_Br_10_], and (c) [TMPA]_2_[Cu_4_Br_6_]. Differences between the measured and calculated patterns are shown in blue.

[TMPA]_2_[Cu_2_Br_4_] has a 0D structure in which isolated dimeric [Cu_2_Br_4_]^2–^ anions are surrounded by TMPA^+^ organic cations. Each Cu­(I) atom is coordinated with three bromine ions in an approximately trigonal planar geometry (Figure 2). In this structure, two adjacent [CuBr_3_]^−^ units share a common edge to form a [Cu_2_Br_4_]^2–^ dimer. From the structural analysis, the observed distance between the two nearest [Cu_2_Br_4_]^2–^ dimers is 5.776 Å. This value is longer than twice of the bromide anion radius (1.96 Å × 2 = 3.92 Å), suggesting negligible interactions between the dimers. The Cu–Br bond distances range from 2.309(8) to 2.426(9) Å, and the Br–Cu–Br angles range from 111.16(3)° to 124.66(3)° within the [Cu_2_Br_4_]^2–^ dimers; these value ranges are indicative of deviations from the ideal trigonal planar geometry. Similar anionic [Cu_2_Br_4_]^2–^ dimers are present in [N­(C_2_H_5_)_4_]_2_[Cu_2_Br_4_],^20,29^ in which the Cu–Br bond distances and Br–Cu–Br angles vary from 2.319(2) Å to 2.454(2) Å and 106.3(1)° to 127.9(1)°, respectively. Yet another compound, [P­(C_2_H_5_)_4_]_2_[Cu_2_Br_4_] has also identical dimeric anions with similar Cu–Br bond distances and Br–Cu–Br angles ranging from 2.263(4) Å to 2.436(3) Å and 107.6(1)° to 128.2(1)°.^17^ In all cases, bond distances and angles are nearly identical, and they suggest distortion of the Cu­(I) coordination environment. Another important structural parameter is the distance between the two nearest Cu ions. This value is 2.738(2) Å for [TMPA]_2_[Cu_2_Br_4_], whereas the Cu···Cu interatomic distances are 2.937(3) Å and 2.870(5) Å for [N­(C_2_H_5_)_4_]_2_[Cu_2_Br_4_] and [P­(C_2_H_5_)_4_]_2_[Cu_2_Br_4_], respectively.^17,20,29^ It has been reported that distortions of copper halide polyhedra and the Cu···Cu distances in Cu­(I) halides with polynuclear anionic units significantly influence the photophysical properties.^1,6,17^ Furthermore, the Cu···Cu distance in the photoexcited state is a key factor to consider. This distance can change significantly compared to the ground state.^1,6^ For example, the recently reported compound [P­(C_2_H_5_)_4_]_2_[Cu_2_Br_4_] demonstrates a 12% shortening of the Cu···Cu distances in the photoexcited state.^6^ This change facilitates the formation of highly stable STEs at room temperature.^6^ It also leads to midgap emission with an almost unity photoluminescence quantum yield (PLQY).^6^ However, such studies, where changes in Cu···Cu distances between the ground and excited states of Cu­(I) halides are observed and linked to their optical properties, are relatively rare. Moreover, several factors can influence the photophysical properties of these materials, including the trapping depth of STEs, the Cu­(I) coordination environment, and the nature of the organic cation.^6,19^ Establishing a direct relationship between Cu···Cu distances and optical properties is complex and requires more systematic studies. Nevertheless, note that the Cu···Cu distance is indeed a critical parameter that can significantly affect photophysical behavior. Identifying an optimal Cu···Cu distance may help in the design of highly efficient Cu­(I) halides, paving the way for customized materials for future practical applications.

The second compound, [TMPA]_4_[Cu_6_Br_10_], also has a 0D structure based on trigonal planar [CuBr_3_]^2–^ anions as the basic structural building units. In this case, six trigonal planar [CuBr_3_]^2–^ units are further interconnected through corner-sharing, forming a unique [Cu_6_Br_10_]^4–^ cluster. The [Cu_6_Br_10_]^4–^ clusters are isolated by the surrounding [TMPA]^+^ cations, forming a 0D structure. In this structure, the [Cu_6_Br_10_]^4–^ clusters are well separated with the nearest Br···Br separation distance of 7.626 Å; this value is significantly larger than the separation between the anion clusters in [TMPA]_2_[Cu_2_Br_4_], suggesting even greater charge localization in the case of [TMPA]_4_[Cu_6_Br_10_]. Although this compound was structurally characterized before,^30^ to the best of our knowledge, photophysical properties of compounds containing [Cu_6_Br_10_]^4–^ clusters have not yet been reported and the impact of the greater charge localization observed in [TMPA]_4_[Cu_6_Br_10_] on its optical properties is yet to be studied. In this compound, the Cu–Br bond distances range from 2.3317(19) to 2.488(2) Å, and the Br–Cu–Br angles range from 112.37(7)° to 130.19(8)°, which suggest significant deviations from the ideal trigonal planar geometry in [Cu_6_Br_10_]^4–^ clusters. Each six membered ring contains two close Cu···Cu contacts measured at 2.660(2) and 2.687(2) Å (Figure S3).

Through additional synthesis experiments, the new compound [TMPA]_2_[Cu_4_Br_6_] with a unique 1D structure was obtained. This compound features 1D [Cu_4_Br_6_]^2–^ anionic chains separated by [TMPA]^+^ cations. These anionic chains are composed of two edge-sharing [CuBr_4_]^3–^ tetrahedra, which are tied to each other along the *a*-axis via bridging trigonal planar [CuBr_3_]^2–^ anions (Figure 2c and Figure S4). Interestingly, there have been other Cu­(I) halides reported in the literature containing [Cu_4_Br_6_]^2–^ anions.^17,20,25,27^ However, these are 0D clusters involving edge-sharing trigonal planar [CuBr_3_]^2–^ units, in contrast to the 1D chains made of both tetrahedral [CuBr_4_]^3–^ and trigonal planar [CuBr_3_]^2–^ anions in [TMPA]_2_[Cu_4_Br_6_]. Within the 1D chains, tetrahedral distortions are observed with the Br–Cu–Br angles ranging from 100.91(2)° to 114.90(2)° and the Cu–Br bond distances ranging from 2.4308(6) to 2.5383(6) Å. Such distortions are also common in other reported Cu­(I) halides featuring tetrahedral building blocks.^6,10,17,20,24,25,28^ Similarly, the trigonal planar units demonstrate distortions, deviating from the ideal trigonal planar values, with the Br–Cu–Br angles ranging from 111.31(2)° to 131.89(2)°. The Cu–Br bond distances in [CuBr_3_]^2–^ vary from 2.3568(6) Å to 2.4924(6) Å. These values are typical for hybrid compounds with trigonal planar [CuBr_3_]^2–^ structural units.^17,20,26^

To evaluate the thermal properties of the compounds, simultaneous DSC and TGA measurements were also performed (Figure S5). The results suggest that at elevated temperatures, all compounds decompose through multistep pathways. The initial thermal decomposition temperatures for [TMPA]_2_[Cu_2_Br_4_], [TMPA]_4_[Cu_6_Br_10_], and [TMPA]_2_[Cu_4_Br_6_] were recorded at 121.97 °C, 126.77 °C, and 101.99 °C, respectively. Through further heating, two additional endothermic events were observed, corresponding to the second and third decomposition steps. These events likely arise from the stepwise thermal degradation and evaporation of the organic trimethylphenylammonium bromide ([N­(C_6_H_5_)­(CH_3_)_3_]­Br) component. However, these onset points do not correspond to the melting transitions of these compounds. The melting behavior was examined over a temperature range of 40–320 °C, but no melting transitions were detected within this range. The total observed weight losses for [TMPA]_2_[Cu_2_Br_4_], [TMPA]_4_[Cu_6_Br_10_], and [TMPA]_2_[Cu_4_Br_6_] were 64.057%, 53.790%, and 41.153%, respectively, approximately match the weight of TMPABr (trimethylphenylammonium bromide) in each compound (60.105%, 50.112%, and 42.069%, respectively). This value suggests that the decomposition likely results from thermal degradation and evaporation of the organic structural component.

Diffuse reflectance measurements were conducted on polycrystalline powder samples (Figure 4). Based on these measurements, [TMPA]_2_[Cu_2_Br_4_], [TMPA]_4_[Cu_6_Br_10_], and [TMPA]_2_[Cu_4_Br_6_] exhibit absorption onsets at 3.61 3.11, and 3.06 eV, respectively. These band gaps are very similar to those of other reported Cu­(I) halides containing [Cu_2_Br_4_]^2–^ and [Cu_4_Br_6_]^2–^ anions, which range from 2.71 to 4.0 eV.^17,20,25,26,46^ The results of the DFT calculations are provided in Figure 5. The compounds exhibit direct band gaps of 2.06 1.98, and 1.97 eV for [TMPA]_2_[Cu_2_Br_4_], [TMPA]_4_[Cu_6_Br_10_], and [TMPA]_2_[Cu_4_Br_6_], respectively. These calculated band gaps are significantly lower than the experimentally measured band gaps, which is typical for DFT calculations using the PBE functional. This underestimation arises from the known limitations of the exchange correlation functionals, particularly in hybrid organic–inorganic metal halides.^28,47–51^ Importantly, the electronic band structures reveal that the highest occupied molecular orbitals (HOMO) and the lowest unoccupied molecular orbitals (LUMO) are composed of localized electronic states. [TMPA]_2_[Cu_2_Br_4_] and [TMPA]_4_[Cu_6_Br_10_] show relatively flat valence bands, indicating strong localization, whereas [TMPA]_2_[Cu_4_Br_6_] displays more pronounced band dispersion, particularly along the 1D chain propagation direction (i.e., the *a*-axis). Furthermore, the projected density of states (PDOS) plots reveal that for [TMPA]_2_[Cu_2_Br_4_] and [TMPA]_4_[Cu_6_Br_10_], the valence band maximum (VBM) is mainly composed of Cu d and Br p orbitals, while the conduction band minimum (CBM) is dominated by contributions from the organic component. In contrast, for [TMPA]_2_[Cu_4_Br_6_], the PDOS shows that both the VBM and CBM are primarily derived from the inorganic component, with a slight contribution from the organic component (Figure S6). Overall, the electronic structures of all three compounds indicate a significant contribution from the organic components near the Fermi level, particularly at the CBM, suggesting a type-II band alignment. According to the literature, type-II band alignment leads to quenched PL in hybrid Cu­(I) halides.^1,6,52^ Therefore, our computational results are consistent with the low PLQYs observed for these compounds (vide infra).

**Figure 4:**
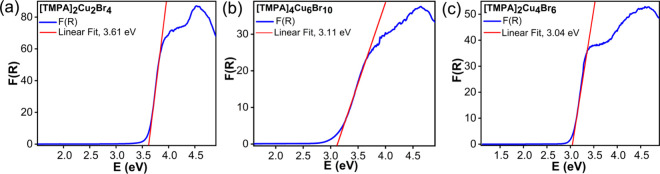
Optical absorption data obtained using the Kubelka–Munk function, F­(R), for (a) [TMPA]_2_[Cu_2_Br_4_], (b) [TMPA]_4_[Cu_6_Br_10_], and (c) [TMPA]_2_[Cu_4_Br_6_].

**Figure 5:**
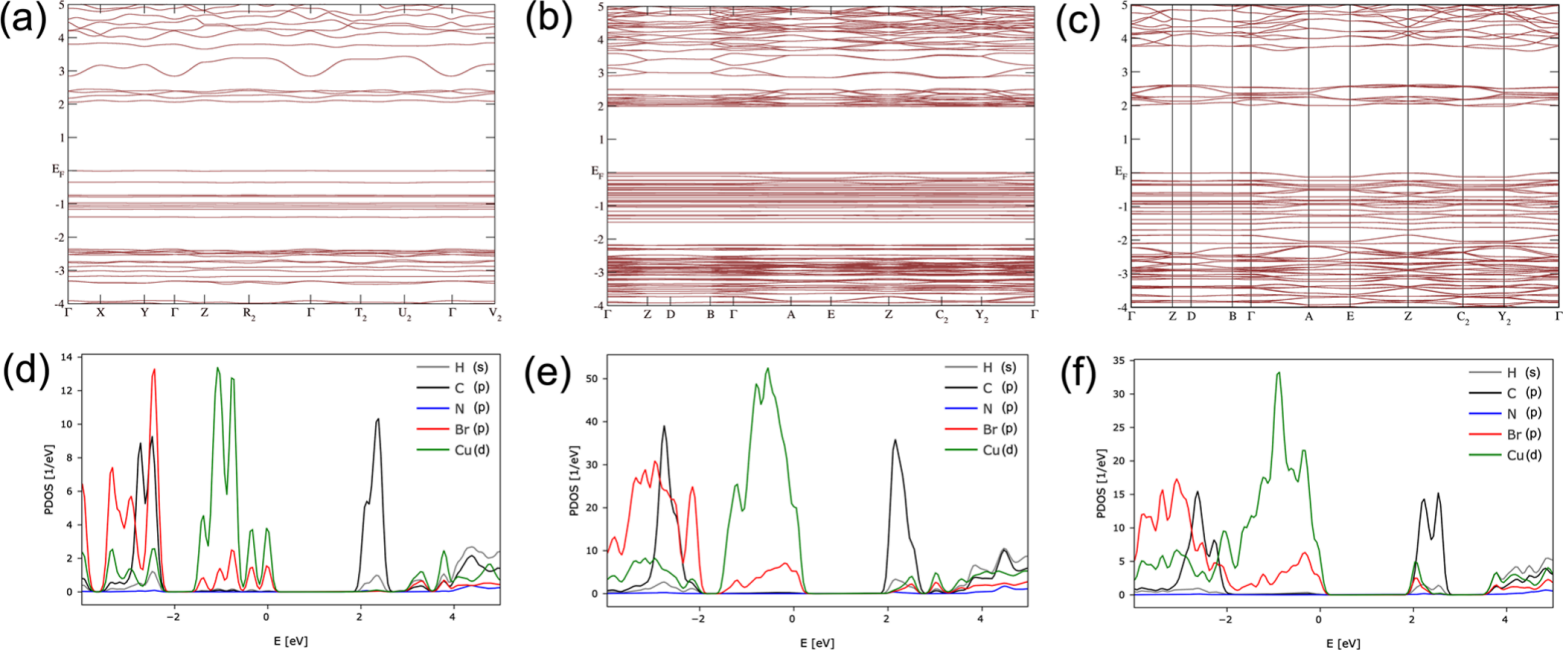
Band structures of (a) [TMPA]_2_[Cu_2_Br_4_], (b) [TMPA]_4_[Cu_6_Br_10_], and (c) [TMPA]_2_[Cu_4_Br_6_]. Projected density of states (PDOS) plots are shown for (d) [TMPA]_2_[Cu_2_Br_4_], (e) [TMPA]_4_[Cu_6_Br_10_], and (f) [TMPA]_2_[Cu_4_Br_6_].

To further understand the optical properties of these materials, PL measurements were carried out. Under daylight, [TMPA]_2_[Cu_2_Br_4_], [TMPA]_4_[Cu_6_Br_10_], and [TMPA]_2_[Cu_4_Br_6_] are colorless and emit blue, orange, and greenish-yellow light, respectively, when illuminated with 365 nm UV light at room temperature. Figure 6 shows the photoluminescence excitation (PLE) and emission (PL) spectra of these compounds. All three compounds display largely Stokes-shifted broad emission peaks, a characteristic feature of STE emission commonly observed in photoluminescent copper­(I) metal halides.^4,13,18,22,24,52–56^

**Figure 6:**
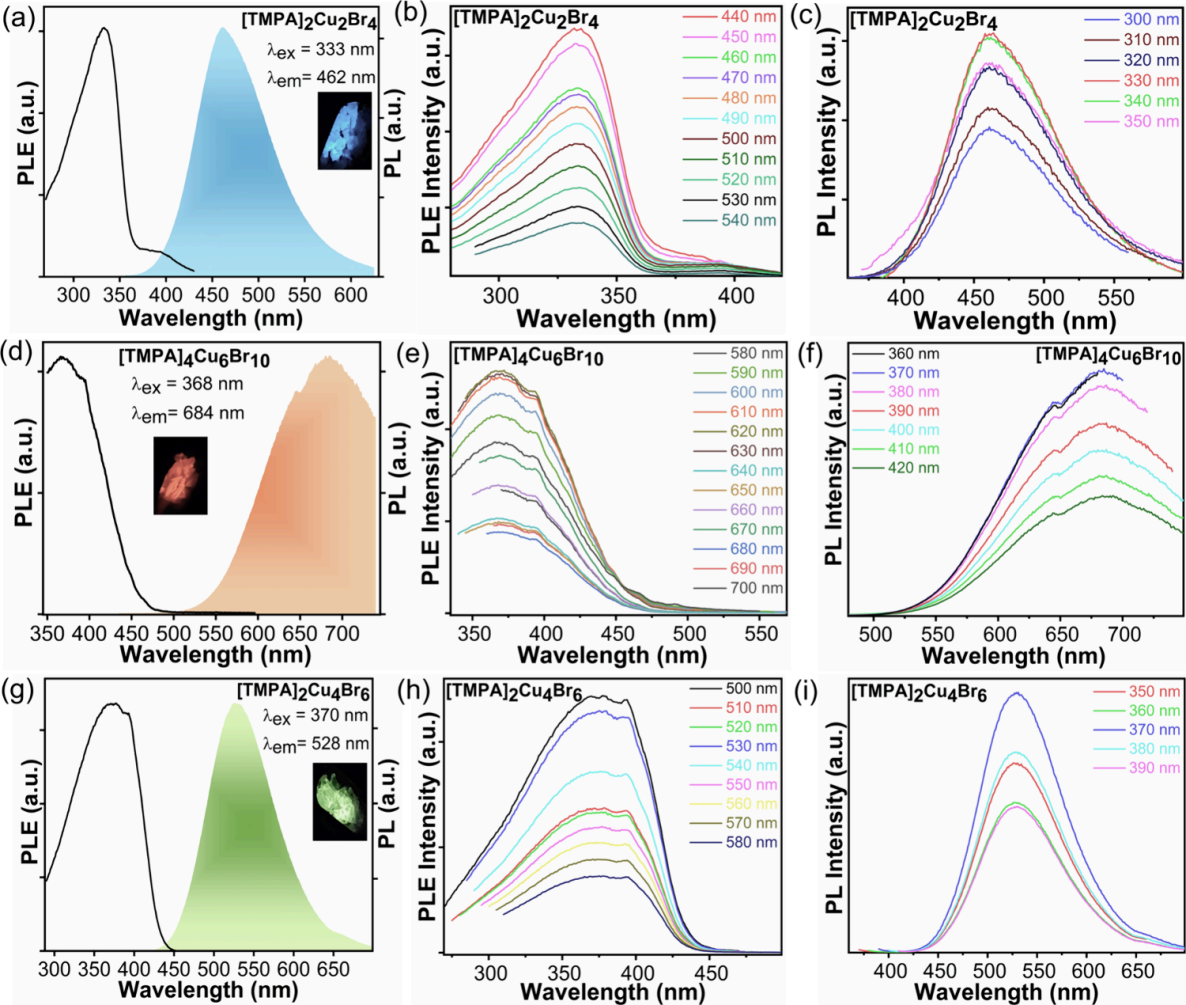
Photoluminescence excitation (PLE) and photoluminescence emission (PL) spectra at room temperature for (a) [TMPA]_2_[Cu_2_Br_4_], (d) [TMPA]_4_[Cu_6_Br_10_], and (g) [TMPA]_2_[Cu_4_Br_6_]. The insets show light emission from the corresponding crystals under UV radiation. Emission-dependent PLE measurements for (b) [TMPA]_2_[Cu_2_Br_4_], (e) [TMPA]_4_[Cu_6_Br_10_], and (h) [TMPA]_2_[Cu_4_Br_6_]. Excitation-dependent PL measurements were for (c) [TMPA]_2_[Cu_2_Br_4_], (f) [TMPA]_4_[Cu_6_Br_10_], and (i) [TMPA]_2_[Cu_4_Br_6_].

[TMPA]_2_[Cu_2_Br_4_] displays an emission band centered at 462 nm when excited at 333 nm with a large Stokes shift of 129 nm. This is comparable to other reported blue-emitting [Cu_2_Br_4_]-based compounds, including [N­(C_2_H_5_)_4_]_2_[Cu_2_Br_4_], [TMEDA]­[Cu_2_Br_4_], [BuDA]­[Cu_2_Br_4_], and [(Me)_4_-Pipz]­[Cu_2_Br_4_], which exhibit PL emissions at 476, 474, 457, and 445 nm, respectively.^20,46^ In contrast, recently reported hybrid compounds containing phosphonium-based organic cations, [P­(C_2_H_5_)_4_]_2_[Cu_2_Br_4_] and [P­(C_2_H_5_)­(C_6_H_5_)_3_]_2_[Cu_2_Br_4_], show greenish-white/yellow emissions with redshifted PL peaks at 503 and 546 nm, respectively.^17,26^ Furthermore, PLQYs of [P­(C_2_H_5_)_4_]_2_[Cu_2_Br_4_] and [N­(C_2_H_5_)_4_]_2_[Cu_2_Br_4_] are very high, 92% and 99.7%, respectively.^17,20,57^ In contrast, [P­(C_2_H_5_)­(C_6_H_5_)_3_]_2_[Cu_2_Br_4_], which has a bulky asymmetric organic cation [P­(C_2_H_5_)­(C_6_H_5_)_3_]^2+^, exhibits a significantly lower PLQY of 22.41%.^26^ To better understand the observed variations in PL efficiencies and composition-structure–property trends, we compare the emission behavior of [TMPA]_2_[Cu_2_Br_4_] with that of other reported [Cu_2_Br_4_]-based compounds (Table S6). Given that previous research suggests Cu···Cu interatomic distances may significantly influence the photophysical properties of luminescent Cu­(I) halides, we examine the Cu···Cu interatomic distances in all of these compounds.^1,17,58^ The observed Cu···Cu interatomic distances for the compounds [N­(C_2_H_5_)_4_]_2_[Cu_2_Br_4_], [P­(C_2_H_5_)_4_]_2_[Cu_2_Br_4_], [P­(C_2_H_5_)­(C_6_H_5_)_3_]_2_[Cu_2_Br_4_], and [TMPA]_2_[Cu_2_Br_4_] are 2.937(3) Å, 2.870(5) Å, 2.86 Å, and 2.738(2) Å, respectively.^17,20,26^ There seems to be a direct correlation between these distances with emission efficiencies, [N­(C_2_H_5_)_4_]_2_[Cu_2_Br_4_] with its longer Cu···Cu interatomic distances having the highest PLQY (99.7%). On the other hand, [TMPA]_2_[Cu_2_Br_4_] has the smallest Cu···Cu interatomic distance and the lowest light emission efficiency. However, it must be noted that these observations are qualitative in nature, and there are multiple factors that influence the observed photophysical properties of Cu­(I) halides.

A better explanation for the observed PLQY differences can be offered based on our computational studies on [TMPA]_2_[Cu_2_Br_4_]. The band structure of this compound shows a significant contribution of the organic cation to the CBM while the VBM is made of inorganic states, suggesting a type-II band alignment. Literature reports indicate that highly emissive Cu­(I) halides have VBM and CBM dominated by Cu 3d and Cu 4s orbitals, respectively, leading to highly localized emissions from the inorganic [CuX] units.^1,6,17,20^ Indeed, PL emissions are ascribed to the localized emission from the inorganic [Cu_2_Br_4_]^2–^ anions in [P­(C_2_H_5_)_4_]_2_[Cu_2_Br_4_] and [N­(C_2_H_5_)_4_]_2_[Cu_2_Br_4_].^17,20^ In contrast, no computational studies have been reported for [P­(C_2_H_5_)­(C_6_H_5_)_3_]_2_[Cu_2_Br_4_] with a lower PLQY of 22.4%,^26^ but we notice the inclusion of aromatic organic cation in this structure. The presence of conjugated organics in hybrid metal halides can lead to the presence of parasitic midgap organic states (i.e., between [Cu_2_Br_4_] states in this case) that quench PL.^52^ In the present case, [TMPA]_2_[Cu_2_Br_4_] with the type-II band alignment shows a quenched PL emission (PLQY < 1%).^1,17,20,57^ These observations lead to two important conclusions: First, the observed variations in the PL spectra and PLQY of [Cu_2_Br_4_]-based compounds suggest that the important roles of organic cations in the Cu­(I) halides cannot be overlooked. Second, the quenched PL in [TMPA]_2_[Cu_2_Br_4_] is attributed to the type-II band alignment of this compound, which leads to separation of charges on different structural units and the very low efficiency of the through space charge transfer (TSCT) processes in hybrid organic–inorganic Cu­(I) halides.^6^ The latter is important and explains yet another observation, and the PLE spectrum of [TMPA]_2_[Cu_2_Br_4_] shows absorption features extending to ∼430 nm (corresponding to 2.88 eV). This is well below the optical gap of the material and corresponds to very weak absorption transitions between the inorganic states in the VBM and organic states in the CBM (see Figure 5). The PLE maximum of 333 nm corresponding to 3.72 eV is much closer to an estimated band gap of 3.61 eV for [TMPA]_2_[Cu_2_Br_4_] from the diffuse reflectance data.

Single crystals of [TMPA]_4_[Cu_6_Br_10_] demonstrate a broad-band orange emission with a peak at 684 nm when excited at 368 nm, giving an unusually large Stokes shift of 316 nm. This compound also shows a type-II band alignment, and consequently, there is weak absorption extending to ∼475 nm in the PLE spectrum of [TMPA]_4_[Cu_6_Br_10_] (Figure 6d). [TMPA]_2_[Cu_4_Br_6_] displays an emission band centered at 528 nm for excitation at 370 nm with a smaller Stokes shift of 158 nm. Given the unique 1D crystal structure of [TMPA]_2_[Cu_4_Br_6_], comparative analysis with other isostructural Cu­(I) halides cannot be performed. The known examples of Cu_4_Br_6_-based compounds have 0D crystal structures and exhibit orange-red emission such as in the cases of (TEP)_2_Cu_4_Br_6_, (TPA)_2_Cu_4_Br_6_, (TBA)_2_Cu_4_Br_6_, and (PTPP)_2_Cu_4_Br_6_ displaying orange-red emissions at 601, 664, 605, and 610 nm, respectively.^17,20,25,27^ This suggests that PL properties can, to some extent, be predicted based on the polyanionic structures if STEs are localized on the inorganic structural part.^1^ Furthermore, research indicates that the PL emission ranges in hybrid Cu­(I) halides are typically comparable when the inorganic polyanions are the same.^1^ However, variations in the PL emission ranges exist because the emissions originate from midgap STE states.^1^ These differences can be attributed to varying degrees of lattice rigidity or deformability, which are influenced by the specific organic counter cations used in each case. Eventually, this influences the structural distortions in the excited state that trap excitons.^1^ Therefore, while crystal structure analysis can somewhat give a limited prediction of optical properties, it lacks precision.

For a more in-depth study of the emission mechanism, we recorded the excitation and emission wavelength dependence of PL and PLE spectra (Figure 6). All three compounds feature identical PL spectra under varied excitation wavelengths and the PLE spectra also remain unchanged. This clearly indicates that the emissions stem from the recombination of the same excited states. To get a clear visualization, all of the collected spectra are normalized and plotted together (Figure S7). The normalized emission and excitation spectra for all compounds demonstrate the identical shapes and features. Following this, room-temperature power-dependent emission spectra were monitored. The integrated emission intensity fitted to a linear power growth model shows a linear relationship between the emission intensity and excitation power (Figure S8). This rules out the possibility of emission from the permanent midgap defect. In the case of defect-based emission, increasing excitation power gradually saturates the finite number of defects.^18,47,59–61^ To ensure that surface defects are not involved in the emission process, we performed the PL measurements on powdered samples.^18,58,62^ Each single crystal was ground into powder, and the PL spectra were measured under the same conditions as those for the single crystals (Figure S9). A slight decrease in PL intensity was observed for the powdered samples. However, the PL peak positions and shapes remained unchanged. This indicates that the influence of surface defect-based emission in the crystals is negligible. Therefore, based on the optical spectroscopy measurements, the light emission in [TMPA]_2_[Cu_2_Br_4_], [TMPA]_4_[Cu_6_Br_10_], and [TMPA]_2_[Cu_4_Br_6_] is attributed to STEs localized on the inorganic structural units. The presence of organic states between the inorganic states in their band structures leads to a partial charge separation (i.e., holes on the inorganic anions and electrons on the organic cations), leading to their much-reduced emission efficiencies as compared to other high-efficiency light-emitting Cu­(I) halides.

Although their PLQYs are low, the PL tunability of [TMPA]_2_[Cu_2_Br_4_], [TMPA]_4_[Cu_6_Br_10_], and [TMPA]_2_[Cu_4_Br_6_] is attractive for use in various luminescent-based applications, such as anticounterfeiting, information storage, and printing fluorescent patterns on different surfaces. For Cu­(I) halides, it has been shown that various engineering, nanostructuring, and processing techniques can be effective in boosting light emission efficiencies.^63^ Here, we prepared luminescent inks based on the microcrystalline powders of all three compounds in toluene or polymer-based matrices; utilizing these, we succeeded in drawing different photoluminescent patterns on distinct surfaces (i.e., glass, plastic, and black paper) (Figure 7 and Figures S10 and S11). Notably, all three compounds demonstrate their corresponding emission colors brightly in the drawings under 365 nm UV light, which indicates the potential of the title compounds for creating a multicolor pattern on different kinds of surfaces for various luminescent-based applications. To further showcase the versatility and usability of [TMPA]_2_[Cu_2_Br_4_], [TMPA]_4_[Cu_6_Br_10_], and [TMPA]_2_[Cu_4_Br_6_] for anticounterfeiting and information storage, a four-digit encrypted code of “8888” was created on black paper using the luminescent inks prepared from the title compounds and a freshly made nonluminescent ink. Figure 7 shows the codes under (Figure 7c) daylight and (Figure 7f) 365 nm UV light irradiation. The fabricated code looks colorless under daylight, while UV irradiation reveals the hidden codes of “5228”, “2025”, and “8085”, made using orange, blue, and greenish-yellow inks, respectively. The earth-abundant and nontoxic elemental compositions and inexpensive preparation of the hybrid copper­(I) halides investigated in this study are advantageous compared to the commonly used lead-based luminescent inks.^64^

**Figure 7:**
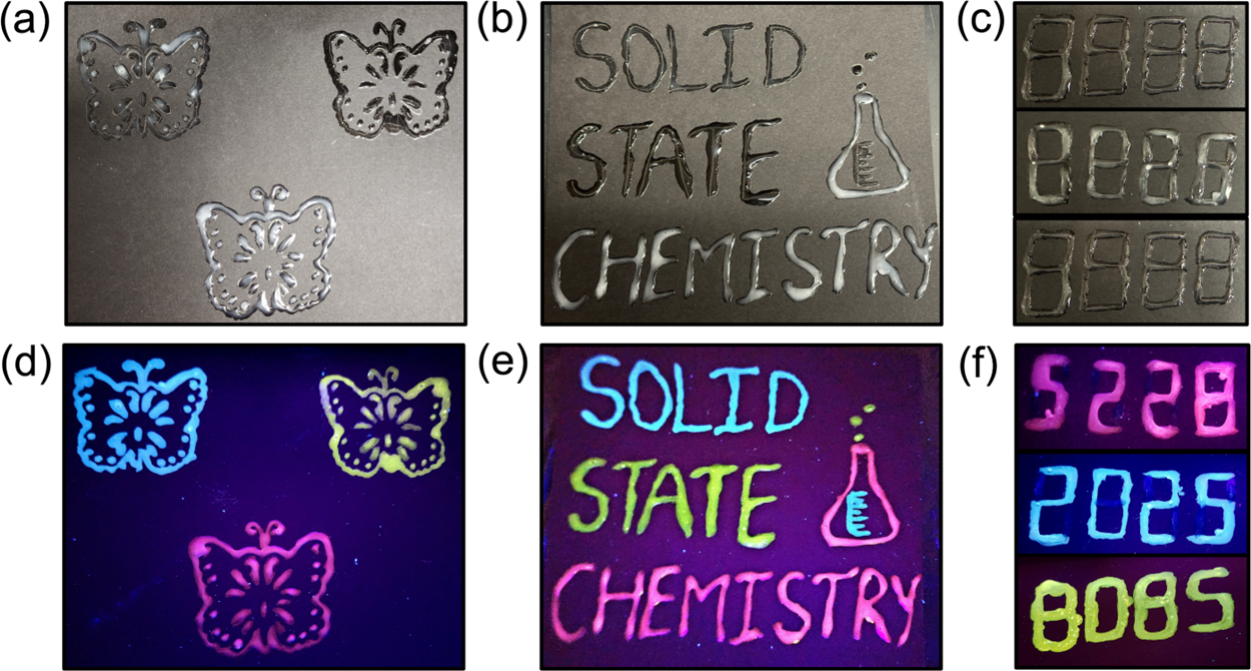
Photographs of (a, d) the printed “Butterfly”, (b, e) “Solid State Chemistry” patterns, and (c, f) encrypted code on black paper using the prepared luminescent inks based on [TMPA]_2_[Cu_2_Br_4_] (blue), [TMPA]_4_[Cu_6_Br_10_] (orange), and [TMPA]_2_[Cu_4_Br_6_] (greenish-yellow) under daylight (top row) and UV light at 365 nm (bottom row).

Recently, stimuli-responsive Cu­(I) halides materials have attracted significant interest for advanced anticounterfeiting and information storage applications.^17,25,26,28^ The use of the same starting reactants, TMPABr and CuBr, but resulting in different compositions and crystal structures in [TMPA]_2_[Cu_2_Br_4_] and [TMPA]_4_[Cu_6_Br_10_], triggered us to explore the possibility of structural transformations between these two compounds. Indeed, our further work in this direction revealed that [TMPA]_2_[Cu_2_Br_4_] can be transformed into [TMPA]_4_[Cu_6_Br_10_] by adding water. The blue-emitting [TMPA]_2_[Cu_2_Br_4_] transforms to orange-emitting [TMPA]_4_[Cu_6_Br_10_] in approximately 50 s when in contact with water (under 365 nm UV light) (Figure 8 and Video S1). The conversion was further confirmed through PXRD experiments recorded before and after the addition of water to [TMPA]_2_[Cu_2_Br_4_] crystals (Figure S12). These findings suggest that the luminescent inks based on the title compounds, i.e., [TMPA]_2_[Cu_2_Br_4_], [TMPA]_4_[Cu_6_Br_10_], and [TMPA]_2_[Cu_4_Br_6_], are multipurpose and can be printed on different surfaces, including glass, plastic, and paper. This versatility can open the path for creative applications of these compounds and other hybrid metal halides.

**Figure 8:**
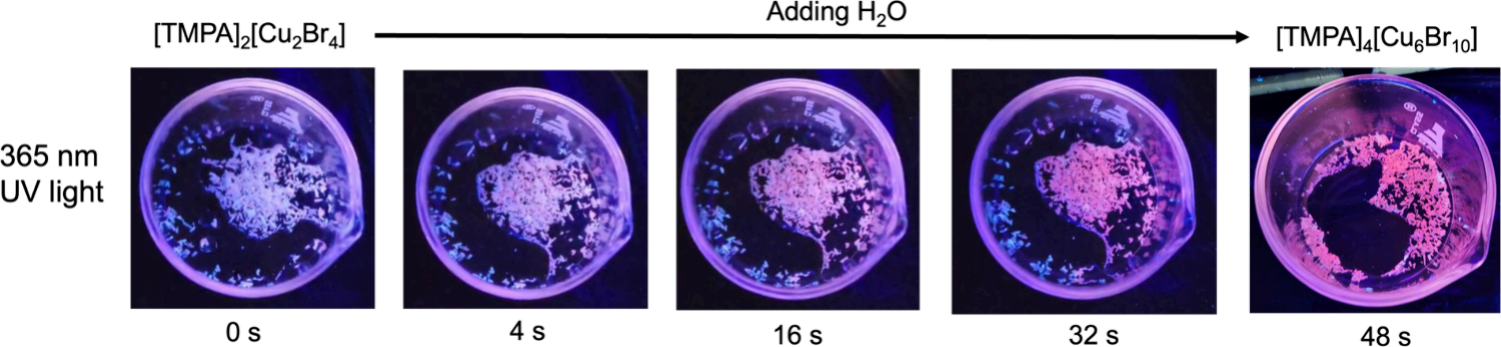
Photographs showing the transformation of [TMPA]_2_[Cu_2_Br_4_] (blue) to [TMPA]_4_[Cu_6_Br_10_] (orange) induced by water addition under 365 nm UV light in approximately 50 s.

## 4 Conclusions

In summary, this study reports the synthesis and physical properties of three hybrid organic–inorganic copper­(I) bromides: [TMPA]_2_[Cu_2_Br_4_], [TMPA]_4_[Cu_6_Br_10_], and [TMPA]_2_[Cu_4_Br_6_]. Among these, [TMPA]_2_[Cu_4_Br_6_] exhibits a unique one-dimensional structure, while [TMPA]_2_[Cu_2_Br_4_] and [TMPA]_4_[Cu_6_Br_10_] demonstrate zero-dimensional cluster structures. [TMPA]_2_[Cu_2_Br_4_], [TMPA]_4_[Cu_6_Br_10_], and [TMPA]_2_[Cu_4_Br_6_] display distinct emission colors of blue, orange, and greenish-yellow, respectively. The studied compounds show negligible PL at room temperature, in contrast to most other hybrid Cu­(I) halides known for remarkably high light emission efficiencies. In latter compounds, the VBM and CBM are primarily derived from Cu 3d and Cu 4s orbitals, respectively.^1,3,6^ However, substantial contribution from the organic cations to the CBM can result in a type-II band alignment, which facilitates nonradiative recombination and suppresses photoluminescence.^1,6^ In our compounds, electronic structure calculations reveal that the presence of organic states between the inorganic states in their band structures leads to a partial charge separation (i.e., holes on the inorganic anions and electrons on the organic cations), accounting for their much-reduced emission efficiencies. These findings highlight the critical role of organic cation selection in the design of light-emitting Cu­(I) halides and the impact of the organic cations on the electronic structure, particularly the orbital character of the band edges.

## Supplementary Material





## Notes

Conversion of [TMPA]_2_[Cu_2_Br_4_] to [TMPA]_4_[Cu_6_Br_10_] (MP4)
Single crystal data and structure refinement parameters for [TMPA]_2_[Cu_2_Br_4_], [TMPA]_4_[Cu_6_Br_10_], and [TMPA]_2_[Cu_4_Br_6_]; atomic coordinates and equivalent isotropic displacement parameters for [TMPA]_2_[Cu_2_Br_4_], [TMPA]_4_[Cu_6_Br_10_], and [TMPA]_2_[Cu_4_Br_6_]; photographs of the synthesized crystals; coordination environment of metals in [TMPA]_2_[Cu_2_Br_4_], [TMPA]_4_[Cu_6_Br_10_], and [TMPA]_2_[Cu_4_Br_6_]; two close Cu···Cu contacts in [TMPA]_4_[Cu_6_Br_10_]; _∞_^1^[Cu_4_Br_6_]^2–^ 1D chain in [TMPA]_2_[Cu_4_Br_6_]; thermogravimetric analysis and differential scanning calorimetry plots for [TMPA]_2_[Cu_2_Br_4_], [TMPA]_4_[Cu_6_Br_10_], and [TMPA]_2_[Cu_4_Br_6_]; band structures, and projected density of states (PDOS) plots for [TMPA]_2_[Cu_4_Br_6_]; normalized PLE and PL spectra for [TMPA]_2_[Cu_2_Br_4_], [TMPA]_4_[Cu_6_Br_10_], and [TMPA]_2_[Cu_4_Br_6_]; room-temperature PL intensity of the emission band versus excitation power for [TMPA]_2_[Cu_2_Br_4_], [TMPA]_4_[Cu_6_Br_10_], and [TMPA]_2_[Cu_4_Br_6_]; a comparison of photoluminescence emission intensity of single crystals and polycrystalline powder samples of [TMPA]_2_[Cu_2_Br_4_], [TMPA]_4_[Cu_6_Br_10_], and [TMPA]_2_[Cu_4_Br_6_]; an illustration of the printed “OU” graffiti on a glass slide, plastic envelope, and black paper using the luminescent ink of [TMPA]_4_[Cu_6_Br_10_] under daylight and 365 nm UV light; pictures of the printed landscape on the glass surface using the luminescent inks of [TMPA]_2_[Cu_2_Br_4_], [TMPA]_4_[Cu_6_Br_10_], and [TMPA]_2_[Cu_4_Br_6_] under daylight and 365 nm UV light; PXRD patterns showing the conversion of [TMPA]_2_[Cu_2_Br_4_] to [TMPA]_4_[Cu_6_Br_10_] after water addition; summary of the photoluminescence properties and Cu···Cu interatomic distances of discrete [Cu_2_Br_4_]^2–^ anion-based hybrid organic–inorganic Cu­(I) bromides (PDF)
