# Supplementary material for: Structural Diversity and Tunable Emission in Hybrid Organic–Inorganic Copper(I) Bromides
Source: Inorg Chem. 2025 Dec 8;64(50):24796–807. doi: 10.1021/acs.inorgchem.5c04578 (PMC12728924; doi:10.1021/acs.inorgchem.5c04578)
Supplement: Supplementary file 2 [file ic5c04578_si_002.pdf]

Supplementary Information

Structural Diversity and Tunable Emission in Hybrid Organic-Inorganic Copper(I) Bromides

Tamanna Pinky[a], Kanika Parashar [a], Dilruba A. Popy[a], Kowsik Ghosh[b], Aleksandra D. Valueva[c], Mario F. Borunda[d], Svilen Bobev[b], Bayram Saparov[a*]

[a]*Department of Chemistry & Biochemistry, University of Oklahoma, Norman, Oklahoma 73019*

[b]*Department of Chemistry & Biochemistry, University of Delaware, Newark, Delaware 19716*

[c]*Department of Chemistry, University of Georgia, Athens, Georgia 30602*

[d]*Department of Physics, Oklahoma State University, Stillwater, Oklahoma 74078*

*Author to whom correspondence should be addressed: saparov@ou.edu

Table S1. Single crystal data and structure refinement parameters for $[TMPA]_2[Cu_2Br_4]$, $[TMPA]_4[Cu_6Br_{10}]$, and $[TMPA]_2[Cu_4Br_6]$.

Formula	$[TMPA]_2[Cu_2Br_4]$	$[TMPA]_4[Cu_6Br_{10}]$	$[TMPA]_2[Cu_4Br_6]$
Formula weight (g/mol)	719.14	862.59	1006.04
Temperature (K)	293(2)		100(2)
Radiation, wavelength (Å)	Mo Kα, 0.71073		
Crystal system	Triclinic	Monoclinic	Monoclinic
Space group	*P*-1	$P2_1/n$	$P2_1/c$
Z	1	4	2
Unit cell parameters	*a* = 7.943(2) Å<br>*b* = 9.146(3) Å<br>*c* = 9.284(3) Å	*a* = 9.546(3) Å<br>*b* = 21.912(9) Å<br>*c* = 12.682(5) Å	*a* = 7.0305(4) Å<br>*b* = 20.6995(12) Å<br>*c* = 9.3659(5) Å
Volume (Å^3)	617.4(3)	2643.9(18)	1362.59(13)
Density (ρ_{calc}) (Mg/m^3)	1.934	2.167	2.452
Absorption coefficient (μ) (mm^{-1})	8.199	9.956	11.887
$\theta_{min} - \theta_{max}$ (°)	3.56 – 25.36	3.17 – 28.39	2.39 – 28.30
Reflections collected	19661	17597	10366
Independent reflections	2187	3635	3389
R^a indices ($I > 2\sigma(I)$)	R_1 = 0.0328<br>wR_2 = 0.0787	R_1 = 0.0510<br>wR_2 = 0.1315	R_1 = 0.0308<br>wR_2 = 0.0618
Goodness-of-fit on F^2	1.084	1.028	1.005
Largest diff. peak and hole (e$^-$/Å^3)	0.656 and -0.610	1.343 and -1.163	0.742 and -0.647

$^aR_1 = \sum||F_0|-|F_c||/\sum|F_0|$; $WR_2 = |\Sigma|w(F_0^2 - F_c^2)^2/\sum|wF_0^{2\,2}|^{1/2}$
where $w = 1/|\sigma^2 F_0^2 + (AP)^2 + BP|$, with $P = (F_0^2 + 2F_c^2)/3$ and weight coefficients *A* and *B*

Table S2. Atomic coordinates and equivalent isotropic displacement parameters ($U_{eq}{}^{a}$) for $[TMPA]_2[Cu_2Br_4]$.

Atom	*x*	*y*	*z*	U_{eq}, Å^2
**$[TMPA]_2[Cu_2Br_4]$**				
Br(1)	0.74948(5)	0.63579(5)	0.72876(4)	0.04561(15)
Br(2)	1.12443(6)	0.96482(5)	0.68502(5)	0.05068(16)
Cu(1)	0.90208(7)	0.86760(6)	0.58671(6)	0.04930(17)
N(1)	0.7662(3)	0.6122(3)	0.2134(3)	0.0321(6)
C(1)	0.6027(4)	0.7023(4)	0.2059(4)	0.0322(7)
C(2)	0.5222(5)	0.7402(4)	0.0734(5)	0.0421(9)
C(3)	0.5391(5)	0.7464(5)	0.3261(5)	0.0479(9)
C(4)	0.8979(5)	0.7126(5)	0.0864(5)	0.0505(10)
C(5)	0.7399(6)	0.4617(5)	0.1894(5)	0.0516(10)
C(6)	0.3719(5)	0.8242(5)	0.0631(6)	0.0565(11)
C(7)	0.8362(6)	0.5705(6)	0.3667(5)	0.0596(12)
C(8)	0.3051(5)	0.8672(5)	0.1833(7)	0.0620(13)
C(9)	0.3874(6)	0.8298(6)	0.3128(6)	0.0637(13)

[a]*U*eq is defined as one-third of the trace of the orthogonalized *Uij* tensor.

Table S3. Atomic coordinates and equivalent isotropic displacement parameters ($U_{eq}{}^{a}$) for $[TMPA]_4[Cu_6Br_{10}]$.

Atom	*x*	*y*	*z*	U_{eq}, Å^2
**$[TMPA]_4[Cu_6Br_{10}]$**				
Br(1)	0.72702(10)	0.63502(5)	0.78260(7)	0.0695(3)
Br(2)	0.17391(10)	0.45896(5)	0.47115(8)	0.0699(3)
Br(3)	0.50413(10)	0.34358(5)	0.50594(8)	0.0726(3)
Br(4)	0.11944(11)	0.25651(5)	0.45129(9)	0.0775(4)
Br(5)	0.47522(12)	0.49331(5)	0.70593(9)	0.0781(3)
Cu(1)	0.65649(16)	0.55659(7)	0.65742(11)	0.0827(4)
Cu(2)	0.27556(15)	0.33222(7)	0.40542(11)	0.0841(4)
Cu(3)	0.40638(15)	0.43848(7)	0.55285(11)	0.0832(4)
N(1)	0.7044(7)	0.8558(3)	0.2804(5)	0.0522(18)
N(2)	0.6163(7)	0.8509(3)	0.7893(5)	0.0511(17)
C(1)	0.4821(8)	0.8440(4)	0.7223(6)	0.0450(19)
C(2)	0.7252(9)	0.9055(4)	0.3603(7)	0.052(2)
C(3)	0.4849(10)	0.8216(4)	0.6222(7)	0.060(2)
C(4)	0.3566(10)	0.8577(5)	0.7619(8)	0.069(3)
C(5)	0.3613(11)	0.8151(5)	0.5602(8)	0.070(3)
C(6)	0.8362(13)	0.9023(5)	0.4321(8)	0.082(3)
C(7)	0.6074(11)	0.8084(5)	0.3202(8)	0.074(3)
C(8)	0.2368(12)	0.8305(5)	0.5976(9)	0.079(3)
C(9)	0.2321(11)	0.8505(5)	0.6989(10)	0.085(3)
C(10)	0.8404(11)	0.8248(5)	0.2618(9)	0.082(3)
C(11)	0.6453(15)	0.8791(6)	0.1762(9)	0.100(4)
C(12)	0.855(2)	0.9483(7)	0.5066(10)	0.116(6)
C(13)	0.767(3)	0.9962(10)	0.5076(15)	0.148(10)
C(14)	0.6319(16)	0.9519(7)	0.3587(14)	0.127(6)
C(15)	0.6638(17)	0.7912(7)	0.8266(17)	0.174(10)
C(16)	0.6012(16)	0.8894(9)	0.8815(15)	0.189(11)
C(17)	0.7278(16)	0.8743(13)	0.7322(13)	0.220(14)
C(18)	C 0.659(3)	0.9990(8)	0.436(2)	0.162(9)

[a]*U*eq is defined as one-third of the trace of the orthogonalized *Uij* tensor.

Table S4. Atomic coordinates and equivalent isotropic displacement parameters ($U_{eq}{}^{a}$) for $[TMPA]_2[Cu_4Br_6]$.

Atom	*x*	*y*	*z*	U_{eq}, Å^2
**$[TMPA]_2[Cu_4Br_6]$**				
Br(1)	0.67189(5)	0.52793(2)	0.65372(4)	0.01576(10)
Br(2)	1.16048(5)	0.62775(2)	0.60646(4)	0.01722(10)
Br(3)	0.81902(5)	0.55915(2)	0.24681(4)	0.01840(10)
Cu(1)	1.32350(7)	0.52452(2)	0.59114(5)	0.02053(13)
Cu(2)	0.92453(7)	0.56466(2)	0.49019(5)	0.02053(13)
N(1)	0.3129(4)	0.64561(14)	1.0718(3)	0.0150(7)
C(1)	0.5865(6)	0.6543(2)	0.9051(4)	0.0180(8)
C(2)	0.4730(5)	0.68235(17)	1.0066(4)	0.0127(7)
C(3)	0.3255(6)	0.6477(2)	1.2328(4)	0.0195(9)
C(4)	0.3056(7)	0.57597(19)	1.0262(5)	0.0227(9)
C(5)	0.4997(5)	0.74632(18)	1.0471(4)	0.0171(8)
C(6)	0.7273(6)	0.6909(2)	0.8435(4)	0.0212(9)
C(7)	0.6390(6)	0.78237(19)	0.9833(4)	0.0211(9)
C(8)	0.1269(6)	0.6757(2)	1.0222(5)	0.0225(9)
C(9)	0.7533(6)	0.7543(2)	0.8817(4)	0.0221(9)

^{a}*U*eq is defined as one-third of the trace of the orthogonalized *Uij* tensor.

Table S5. Selected bond distances and angles within the anionic units in $[TMPA]_2[Cu_2Br_4]$, $[TMPA]_4[Cu_6Br_{10}]$, and $[TMPA]_2[Cu_4Br_6]$.

Atom pair	Distance (Å)	Label	Angle (°)
**$[TMPA]_2[Cu_2Br_4]$**			
Cu—Br(1)	2.3093(8)	Br(1)—Cu—Br(2)	124.05(3)
Cu—Br(2)	2.4243(8)	Br(1)—Cu—Br(2)	124.66(3)
Cu—Br(2)	2.4256(9)	Br(2)—Cu—Br(2)	111.16(3)
Cu—Cu	2.7416(12)	Cu—Br(2)—Cu	68.84(3)
**$[TMPA]_4[Cu_6Br_{10}]$**			
Br(1)—Cu(1)	2.3986(19)	Br(5)—Cu(1)—Br(1)	115.13(7)
Br(1)—Cu(2)	2.488(2)	Br(5)—Cu(1)—Br(2)	130.19(8)
Br(2)—Cu(1)	2.4133(18)	Br(1)—Cu(1)—Br(2)	112.37(7)
Br(2)—Cu(3)	2.4137(19)	Br(4)—Cu(2)—Br(3)	120.15(7)
Br(3)—Cu(3)	2.3746(19)	Br(4)—Cu(2)—Br(1)	119.13(7)
Br(3)—Cu(2)	2.4487(19)	Br(3)—Cu(2)—Br(1)	114.12(7)
Br(4)—Cu(2)	2.3350(18)	Br(5)—Cu(3)—Br(3)	124.74(7)
Br(5)—Cu(3)	2.3317(19)	Br(5)—Cu(3)—Br(2)	116.66(7)
Br(5)—Cu(1)	2.3384(19)	Br(3)—Cu(3)—Br(2)	114.93(7)
Cu(1)—Cu(2)	2.660(2)	Cu(1)—Br(1)—Cu(2)	65.92(6)
Cu(1)—Cu(3)	2.687(2)	Cu(1)—Br(2)—Cu(3)	67.66(6)
		Cu(3)—Br(3)—Cu(2)	82.40(6)
		Cu(3)—Br(5)—Cu(1)	104.54(7)
**$[TMPA]_2[Cu_4Br_6]$**			
Br(1)—Cu(1)	2.5383(6)	Br(1)—Cu(1)—Br(1)	100.91(2)
Br(1)—Cu(1)	2.5055(6)	Br(1)—Cu(1)—Br(3)	106.18(2)
Br(1)—Cu(2)	2.4924(6)	Br(2)—Cu(1)—Br(1)	114.90(2)
Br(2)—Cu(1)	2.4308(6)	Br(2)—Cu(1)—Br(1)	116.48(2)
Br(2)—Cu(2)	2.3568(6)	Br(2)—Cu(1)—Br(3)	111.88(2)
Br(3)—Cu(1)	2.5261(6)	Br(3)—Cu(1)—Br(1)	105.29(2)
Br(3)—Cu(2)	2.3826(6)	Br(2)—Cu(2)—Br(1)	112.91(2)
Cu(1)—Cu(2)	3.0532(7)	Br(2)—Cu(2)—Br(3)	131.89(2)
Cu(1)—Cu(2)	2.6388(6)	Br(3)—Cu(2)—Br(1)	111.31(2)
		Cu(1)—Br(1)—Cu(1)	79.09(2)
		Cu(2)—Br(1)—Cu(1)	124.90(2)
		Cu(2)—Br(1)—Cu(1)	63.266(18)
		Cu(2)—Br(2)—Cu(1)	79.23(2)
		Cu(2)—Br(3)—Cu(1)	64.960(18)

Table S6. Summary of the photoluminescence properties and Cu···Cu interatomic distances of discrete $[Cu_2Br_4]^{2-}$ anion-based hybrid organic-inorganic Cu(I) bromides.

Compound	Cu···Cu	PLE Peak (nm)	PL Peak (nm)	Stokes Shift (nm)	PLQY (%)	Emission Color	Ref.
$[N(C_2H_5)_4]_2[Cu_2Br_4]$	2.937(3)	320	468	148	99.7	Blue	[1, 2]
$[P(C_2H_5)_4]_2[Cu_2Br_4]$	2.870(5)	332	503	171	92	Greenish-white	[3]
$[P(C_2H_5)(C_6H_5)_3]_2Cu_2Br_4$	2.86	279	546	267	22.4	Greenish-yellow	[4]
$[N(C_6H_5)(CH_3)_3]_2[Cu_2Br_4]$	2.738(2)	333	462	129	<1	Blue	This work

(a)

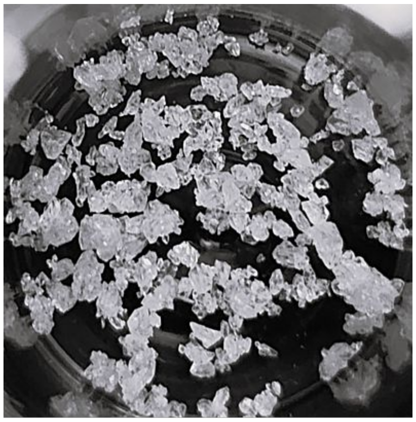

(b)

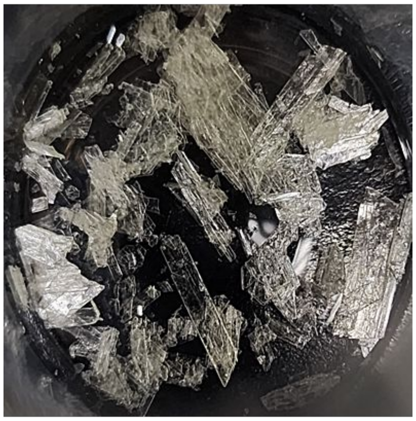

(c)

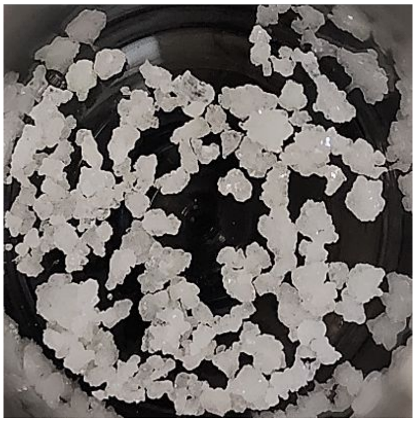

Figure S1. As-synthesized crystals of (a) $[TMPA]_2[Cu_2Br_4]$, (b) $[TMPA]_4[Cu_6Br_{10}]$, and (c) $[TMPA]_2[Cu_4Br_6]$.

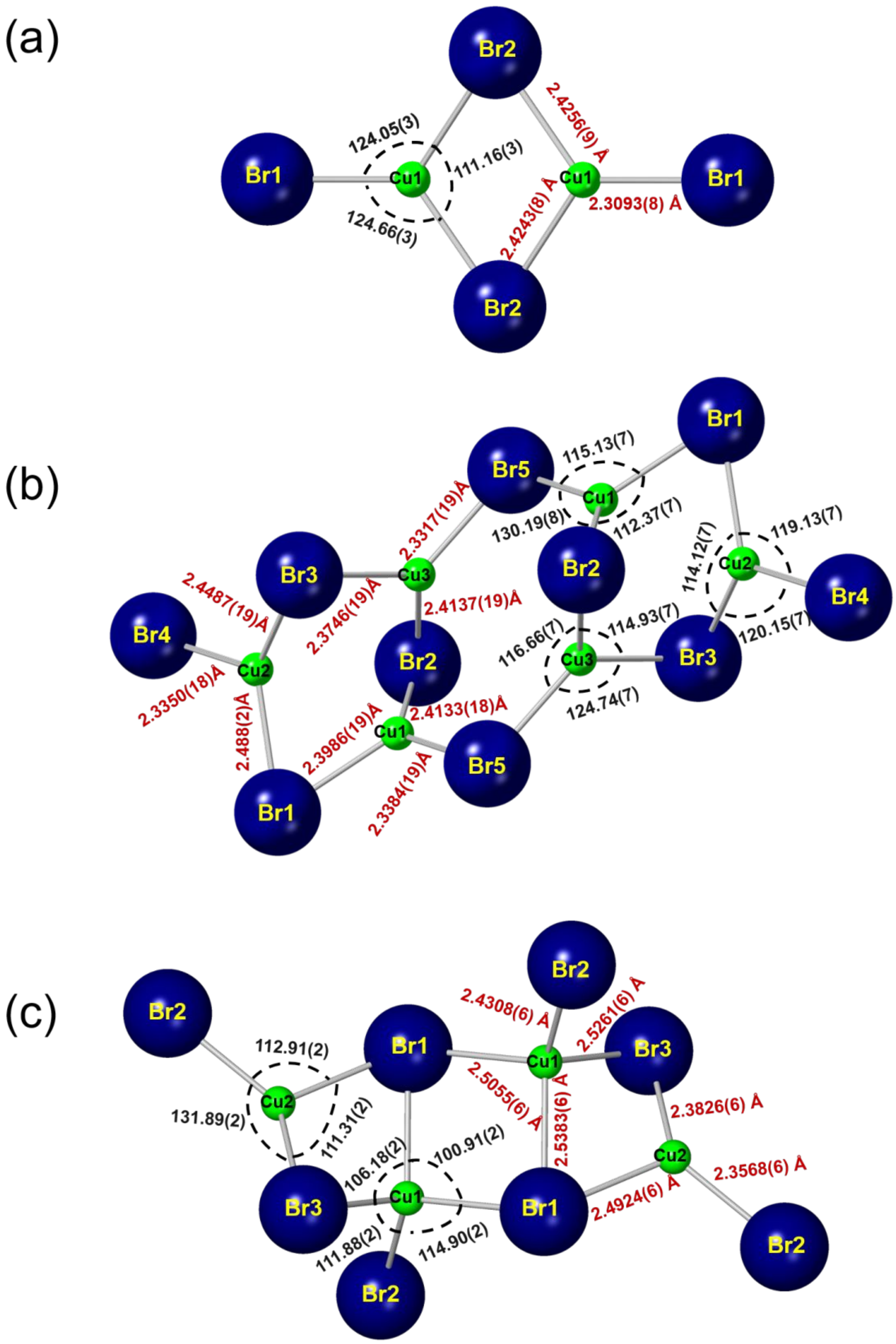

Figure S2. The coordination environments of Cu(I) in (a) $[TMPA]_2[Cu_2Br_4]$, (b) $[TMPA]_4[Cu_6Br_{10}]$, and (c) $[TMPA]_2[Cu_4Br_6]$. Green and blue spheres represent Cu and Br, respectively.

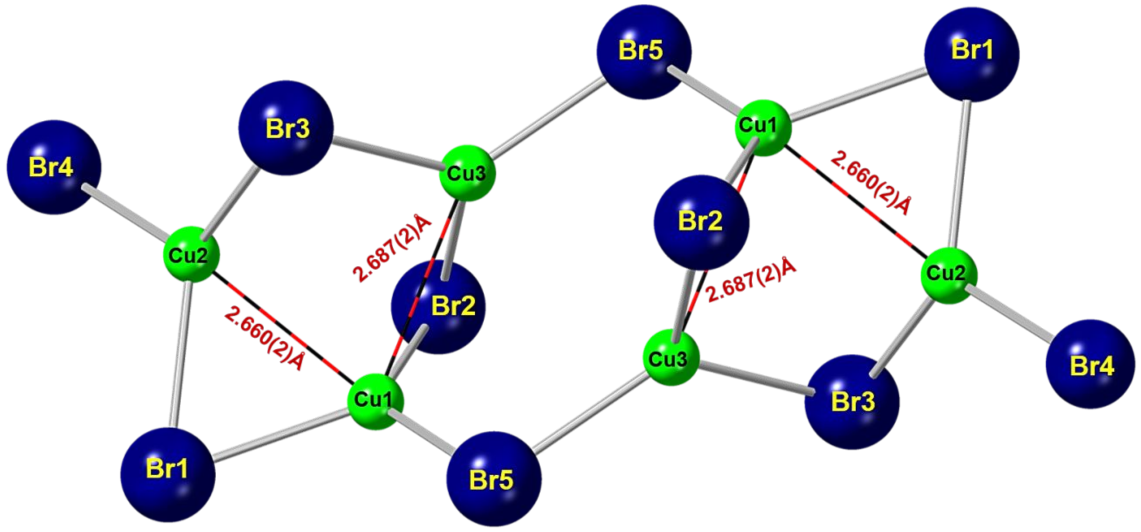

Figure S3. Two close Cu···Cu contacts in $[TMPA]_4[Cu_6Br_{10}]$.

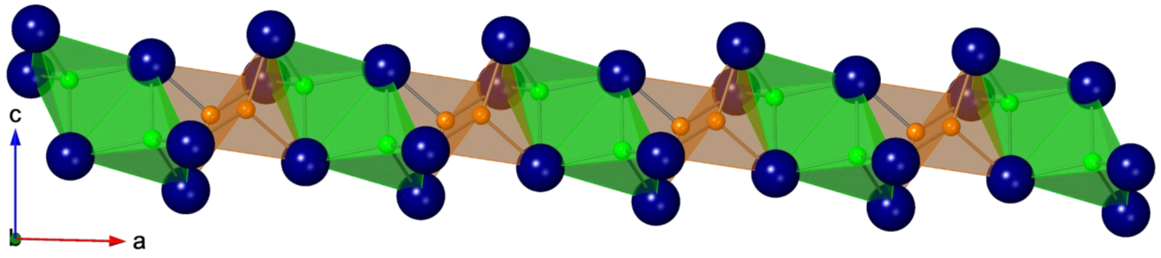

Figure S4. A polyhedral representation of the ${}^{1}_{\infty}[\mathrm{Cu_4Br_6}]^{2-}$ 1D chain in $[TMPA]_2[Cu_4Br_6]$, showing edge-sharing $[CuBr_4]^{3-}$ tetrahedra (green) linked along the a-axis by bridging trigonal planar $[CuBr_3]^{2-}$ units (orange).

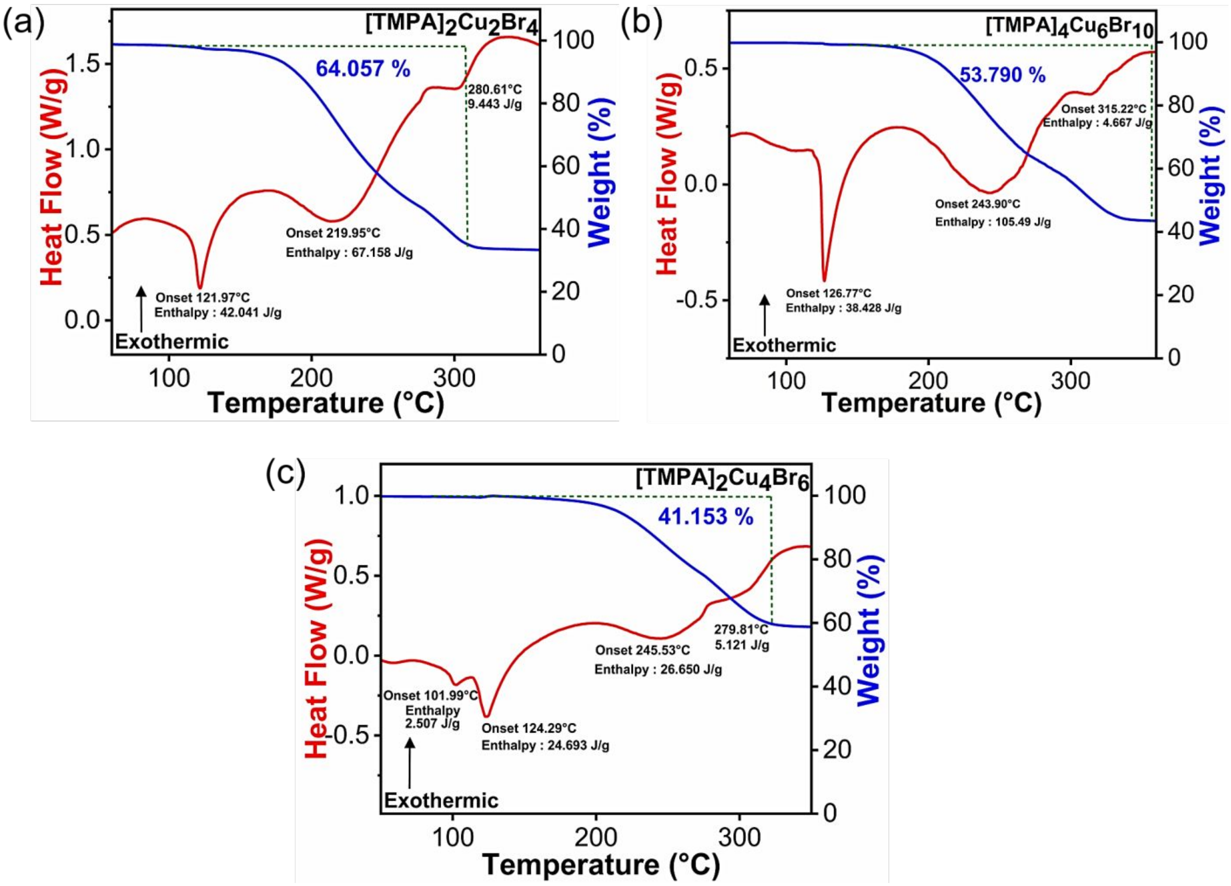

Figure S5. Thermogravimetric Analysis (TGA, in blue) and Differential Scanning Calorimetry (DSC, in red) plots for (a) $[TMPA]_2[Cu_2Br_4]$, (b) $[TMPA]_4[Cu_6Br_{10}]$, and (c) $[TMPA]_2[Cu_4Br_6]$.

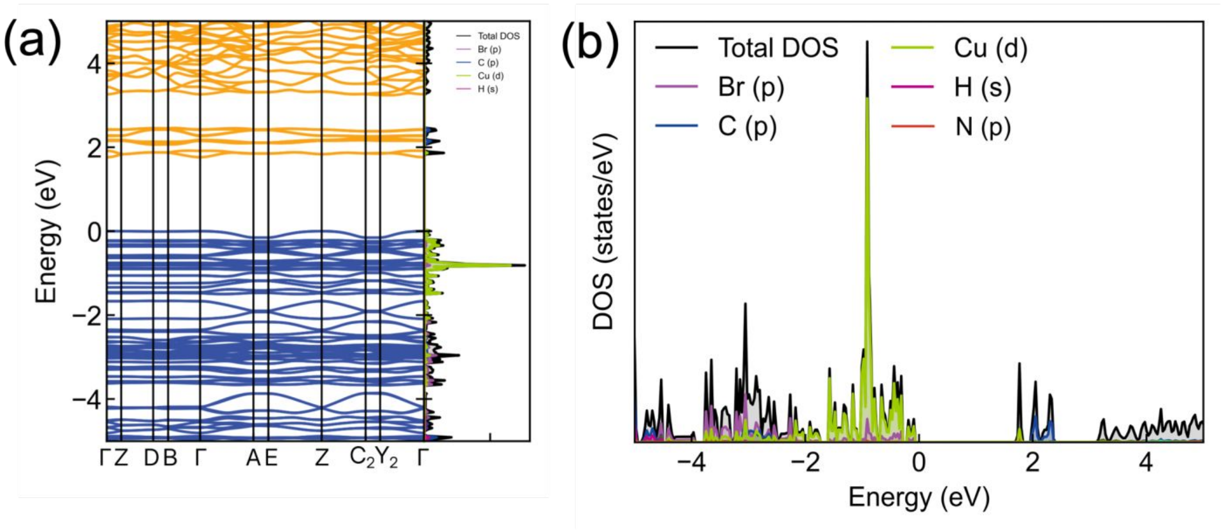

Figure S6. (a) Band structure and for (b) Density of States (DOS) plots for $[TMPA]_2[Cu_4Br_6]$.

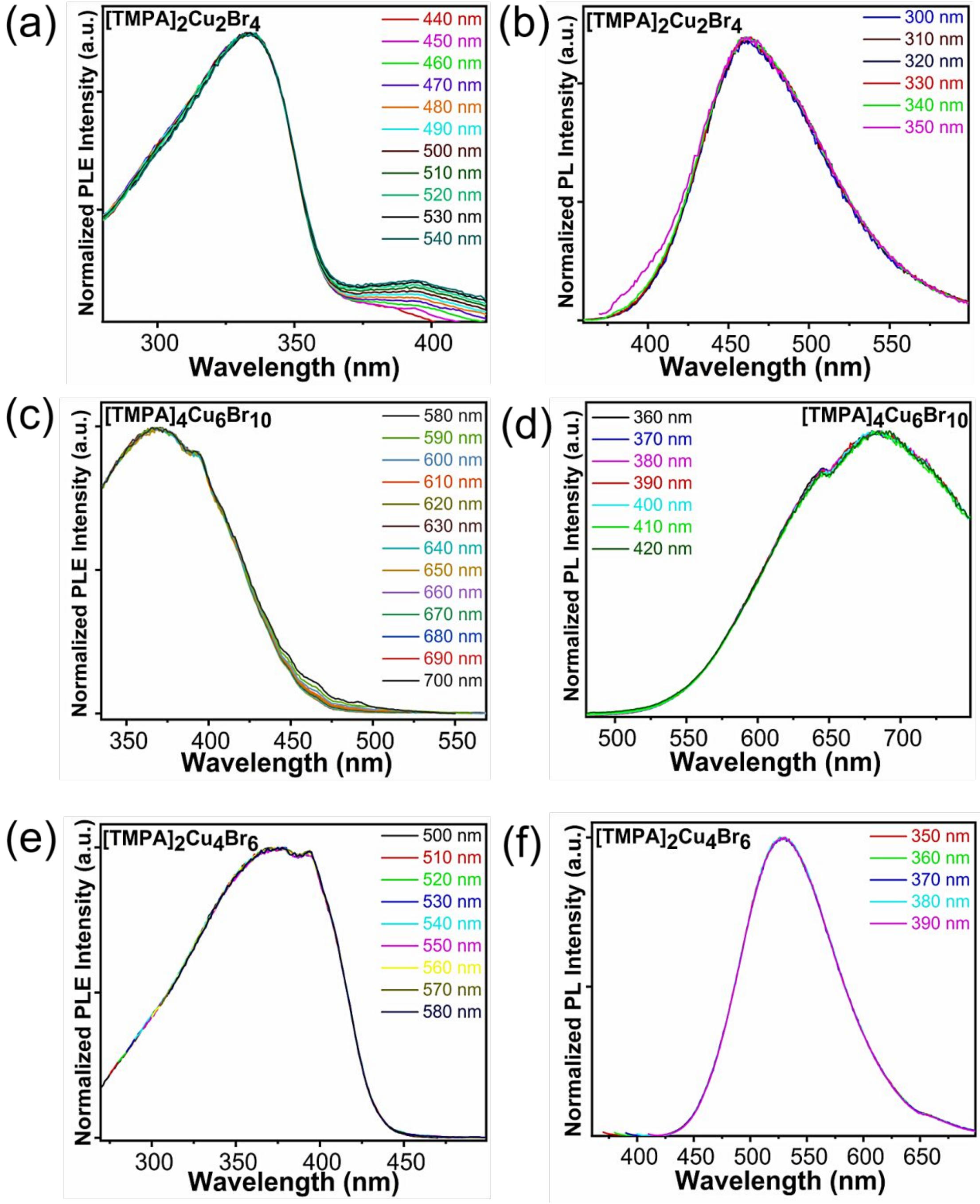

Figure S7. (a) Normalized PLE spectra of $[TMPA]_2[Cu_2Br_4]$ at various emission wavelengths (440-540 nm). (b) Normalized PL spectra of $[TMPA]_2[Cu_2Br_4]$ at various excitation wavelengths (300-350 nm). (c) Normalized PLE spectra of $[TMPA]_4[Cu_6Br_{10}]$ at various emission wavelengths (580-700 nm). (d) Normalized PL spectra of $[TMPA]_4[Cu_6Br_{10}]$ at various excitation wavelengths (360-420 nm). (e) Normalized PLE spectra of $[TMPA]_2[Cu_4Br_6]$ at various emission wavelengths (500-580 nm). (f) Normalized PL spectra of $[TMPA]_2[Cu_4Br_6]$ at various excitation wavelengths (350-390 nm).

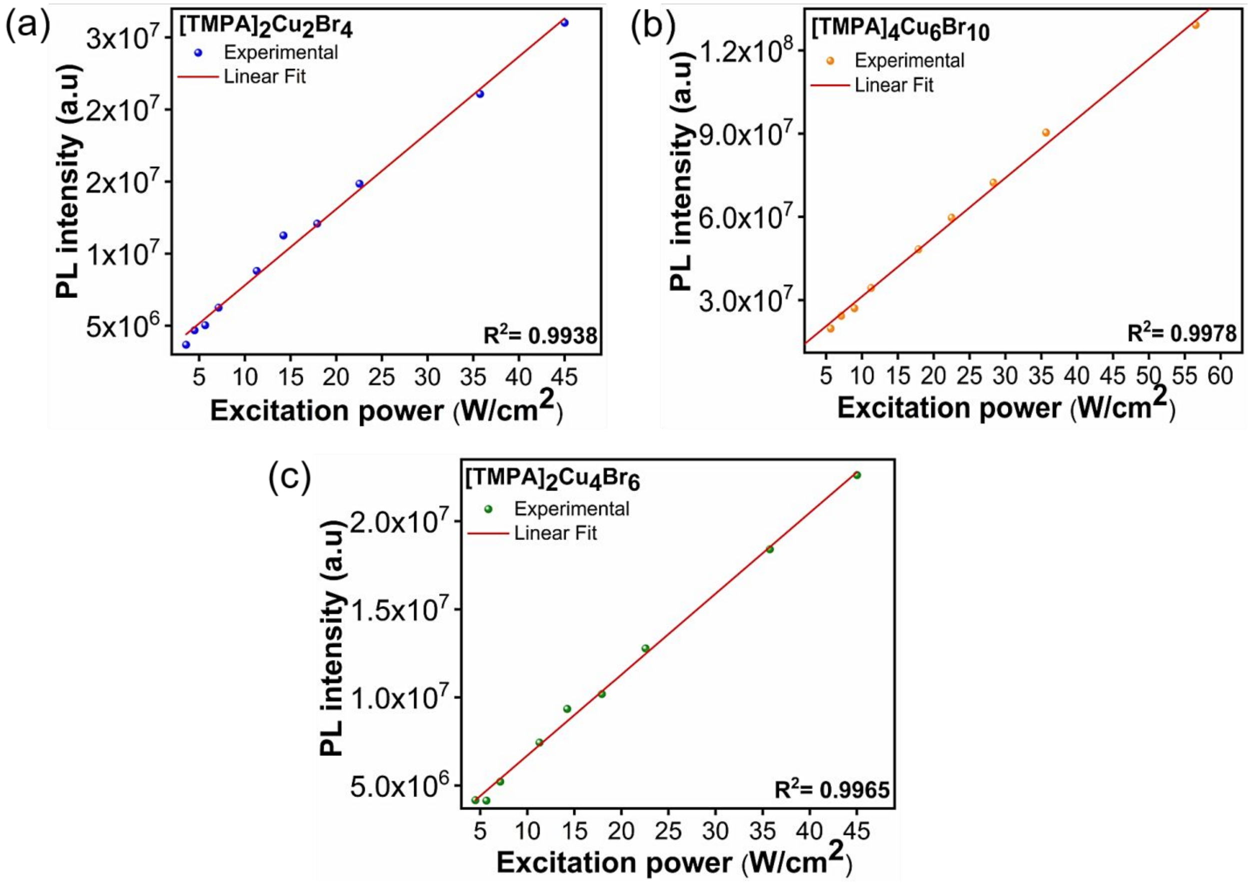

Figure S8. Room temperature PL intensity of the emission band versus excitation power for (a) $[TMPA]_2[Cu_2Br_4]$, (b) $[TMPA]_4[Cu_6Br_{10}]$, and (c) $[TMPA]_2[Cu_4Br_6]$.

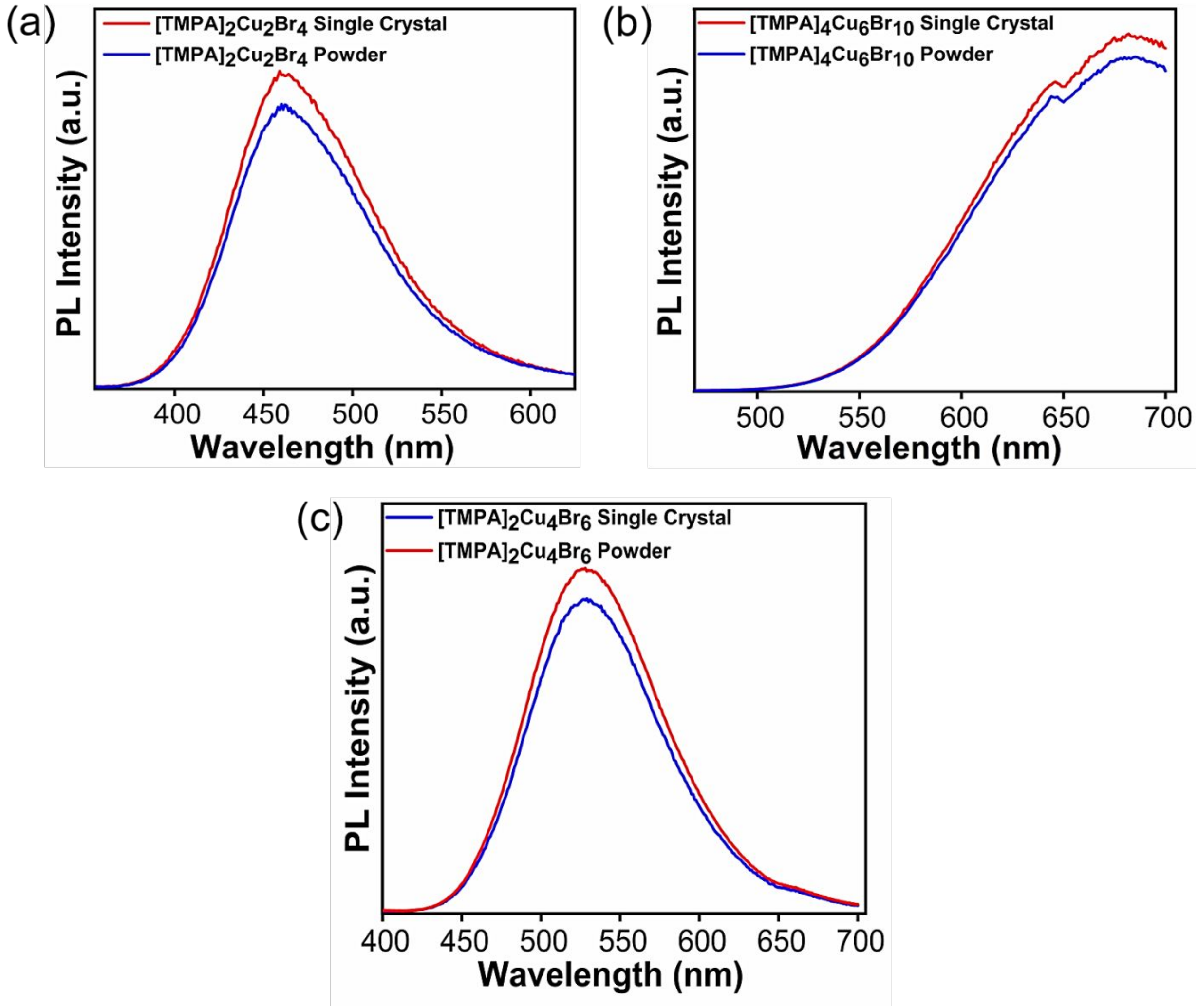

Figure S9. A comparison of photoluminescence emission spectra of single crystals and polycrystalline powder samples of (a) $[TMPA]_2[Cu_2Br_4]$, (b) $[TMPA]_4[Cu_6Br_{10}]$, and (c) $[TMPA]_2[Cu_4Br_6]$.

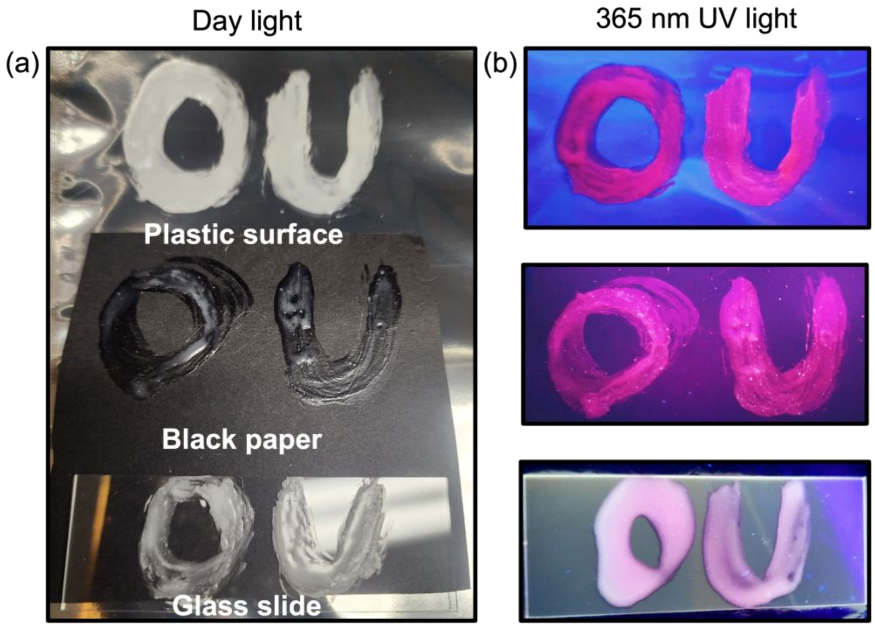

Figure S10. An illustration of the printed 'OU' graffiti on a glass slide, plastic envelope, and black paper using the luminescent ink of $[TMPA]_4[Cu_6Br_{10}]$ (orange) under (a) daylight and (b) 365 nm UV light.

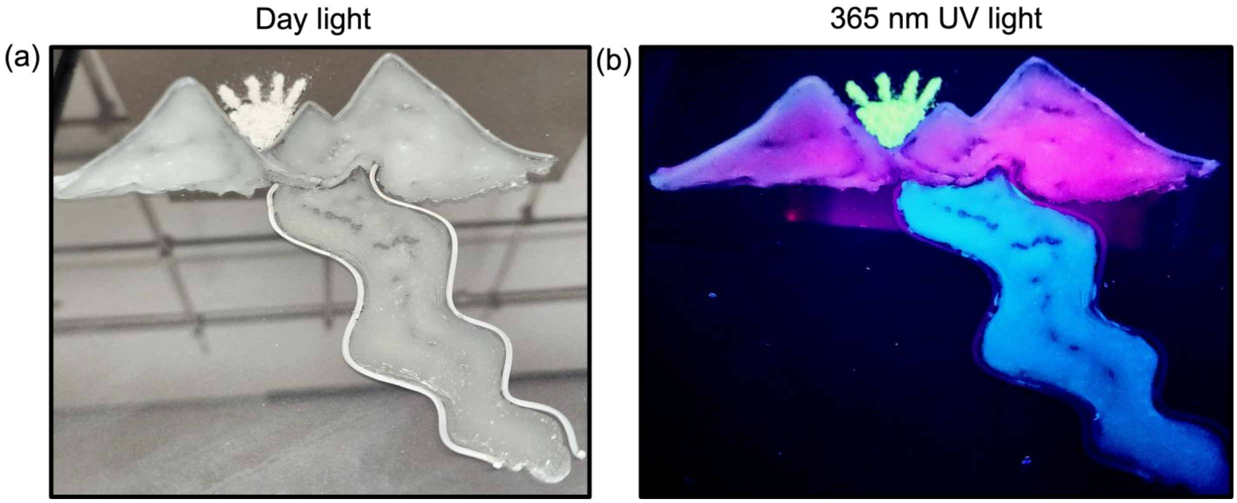

Figure S11. Pictures of the printed landscape on the glass surface using the luminescent inks of $[TMPA]_2[Cu_2Br_4]$ (blue), $[TMPA]_4[Cu_6Br_{10}]$ (orange), and $[TMPA]_2[Cu_4Br_6]$ (greenish yellow) under (a) daylight and (b) 365 nm UV light.

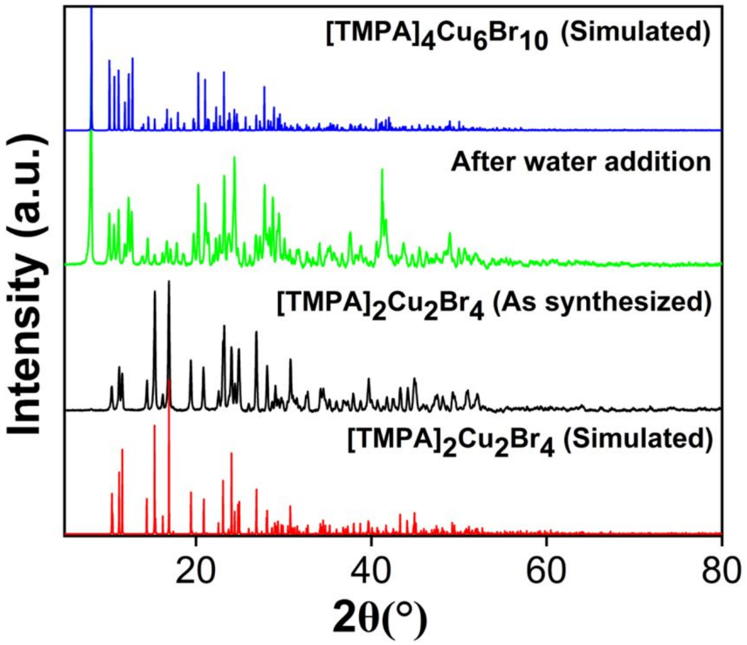

Figure S12. PXRD patterns showing the conversion of $[TMPA]_2[Cu_2Br_4]$ to $[TMPA]_4[Cu_6Br_{10}]$ after water addition.

References

(1) Su, B.; Jin, J.; Han, K.; Xia, Z. Ceramic wafer scintillation screen by utilizing near-unity blue-emitting lead-free metal halide $(C_8H_{20}N)_2Cu_2Br_4$. *Advanced Functional Materials* **2023**, *33* (5), 2210735.

(2) Chen, S.; Gao, J.; Chang, J.; Li, Y.; Huangfu, C.; Meng, H.; Wang, Y.; Xia, G.; Feng, L. Family of highly luminescent pure ionic copper(I) bromide based hybrid materials. *ACS applied materials & interfaces* **2019**, *11* (19), 17513-17520.

(3) Popy, D. A.; Singh, Y.; Tratsiak, Y.; Cardoza, A. M.; Lane, J. M.; Stand, L.; Zhuravleva, M.; Rai, N.; Saparov, B. Stimuli-responsive photoluminescent copper (I) halides for scintillation, anticounterfeiting, and light-emitting diode applications. *Aggregate* **2024**, *5* (5), e602.

(4) Wu, J.; Qi, J.-L.; Guo, Y.; Yan, S.; Liu, W.; Guo, S.-P. Reversible tri-state structural transitions of hybrid copper (I) bromides toward tunable multiple emissions. *Inorganic Chemistry Frontiers* **2024**, *11* (1), 156-163.
